# Genetically Engineering the Nervous System with CRISPR-Cas

**DOI:** 10.1523/ENEURO.0419-19.2020

**Published:** 2020-03-11

**Authors:** Alfredo Sandoval, Hajira Elahi, Jonathan E. Ploski

**Affiliations:** School of Behavioral and Brain Sciences and the Department of Molecular and Cell Biology, University of Texas at Dallas, Richardson, TX 75080

**Keywords:** brain, Cas, Cpf1, CRISPR, nervous system, neuron

## Abstract

The multitude of neuronal subtypes and extensive interconnectivity of the mammalian brain presents a substantial challenge to those seeking to decipher its functions. While the molecular mechanisms of several neuronal functions remain poorly characterized, advances in next-generation sequencing (NGS) and gene-editing technology have begun to close this gap. The clustered regularly interspaced short palindromic repeats (CRISPR)-associated protein (CRISPR-Cas) system has emerged as a powerful genetic tool capable of manipulating the genome of essentially any organism and cell type. This technology has advanced our understanding of complex neurologic diseases by enabling the rapid generation of novel, disease-relevant *in vitro* and transgenic animal models. In this review, we discuss recent developments in the rapidly accelerating field of CRISPR-mediated genome engineering. We begin with an overview of the canonical function of the CRISPR platform, followed by a functional review of its many adaptations, with an emphasis on its applications for genetic interrogation of the normal and diseased nervous system. Additionally, we discuss limitations of the CRISPR editing system and suggest how future modifications to existing platforms may advance our understanding of the brain.

## Visual Abstract

**Figure:**
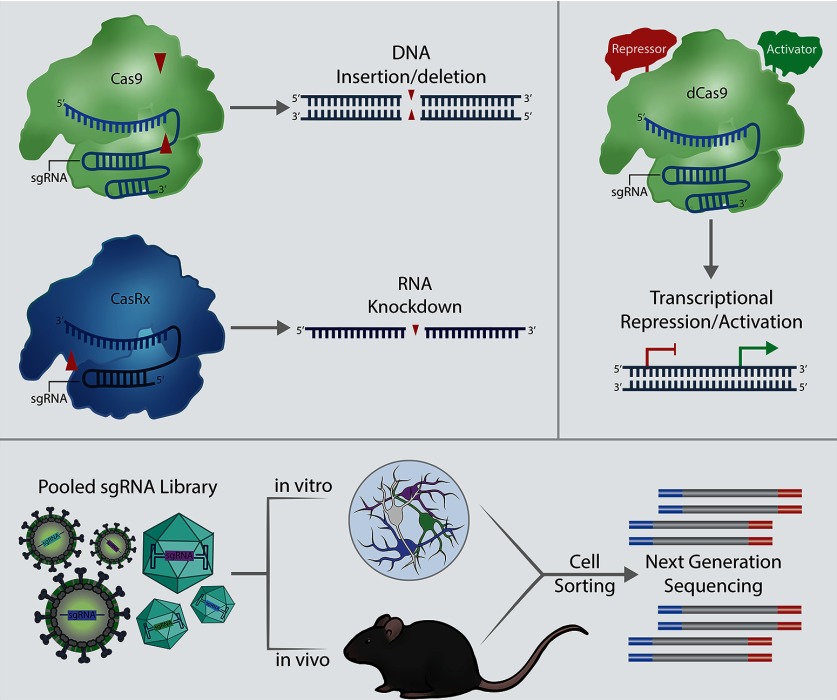

## Introduction

Complex behavior is driven by extensive structural and genetic interactions in the mammalian CNS. Historically, neuroscientists have examined these interactions using a variety of histological, electrophysiological and pharmacological techniques. While indispensable, these techniques lack the specificity of targeted genetic approaches to dissect neuronal function. Recent advances have allowed the coupling of high-throughput next-generation sequencing (NGS) technologies with the cell-type specificity of modern molecular genetics to interrogate complex network interactions and behaviors at unprecedented scale and resolution. The ability to read, write, and manipulate genomes with cell-type specificity is critical, especially considering the cellular heterogeneity of various CNS structures (Chung et al., 2005).

Early attempts at targeted gene editing were performed with zinc finger nucleases (ZFNs) and transcription activator-like effector nucleases (TALENs), both of which rely on programmable DNA-binding proteins coupled to active endonucleases to cleave specific DNA sequences (Kim et al., 1996; Carroll, 2011; Joung and Sander, 2013). While suitable for a variety of applications (Gaj et al., 2013), these systems have fallen out of favor for new genome editing systems due to relative disadvantages such as their extensive protein engineering requirements. Recent advances in gene editing technology have culminated in the discovery of clustered regularly interspaced palindromic repeats (CRISPR)-Cas9, a bacterial immune system which has been repurposed for mammalian genome editing applications (Jinek et al., 2012). Unlike its predecessors, CRISPR nucleases target DNA in an RNA-directed manner, using a programmable single guide RNA (sgRNA) to target complementary DNA sequences for cleavage.

Since the initial adaptation of CRISPR, novel variants continue to be discovered in diverse microbial species, differing in endonuclease size, substrate preference and target recognition requirements (Ran et al., 2015; Abudayyeh et al., 2017). Moreover, several nuclease variants have been engineered for expanded targeting capacity and improved fidelity (Kleinstiver et al., 2015, 2016; Slaymaker et al., 2016; Chen et al., 2017). Perhaps most versatile are the catalytically inactive variants designed to function as DNA-binding proteins, which can regulate transcription, modify the epigenome, target RNA for destruction and facilitate base-editing through the action of their coupled enzymatic domains (Dominguez et al., 2016; Rees and Liu, 2018; Pickar-Oliver and Gersbach, 2019). The highly flexible and multifunctional character of this platform has established CRISPR-Cas as the predominant genome editing system in use today. Here, we provide an overview of CRISPR-Cas technology, followed by a review of its many adaptations for genetic interrogation and modification. Throughout this article, we emphasize applications of CRISPR systems in the field of neuroscience and discuss the potential of this technology to advance our understanding of the brain.

## CRISPR-Cas

Isolated from *Streptococcus pyogenes*, the Type II CRISPR-Cas9 system (spCas9) was the first enzyme repurposed from its native role as a bacterial adaptive immune system for genome editing applications in eukaryotic cells (Jinek et al., 2012). While spCas9 remains the most popular CRISPR nuclease, various CRISPR-Cas systems with divergent structures and properties have been discovered. These systems are broadly categorized by their nuclease composition, with those containing multisubunit nuclease structures pertaining to Class 1 and those composed of a single protein pertaining to Class 2. Within Class 2, systems are further subdivided into Types II, V, and VI, which pertain to DNA-targeting Cas9 and Cas12a and RNA-targeting Cas13, respectively (Shmakov et al., 2017). As Class 2 systems have been used in the majority of neuronal gene editing experiments, they will therefore be the focus of this review. Class 1 systems and their uses are described elsewhere (Cameron et al., 2019; Pickar-Oliver et al., 2019).

**Figure 1.**
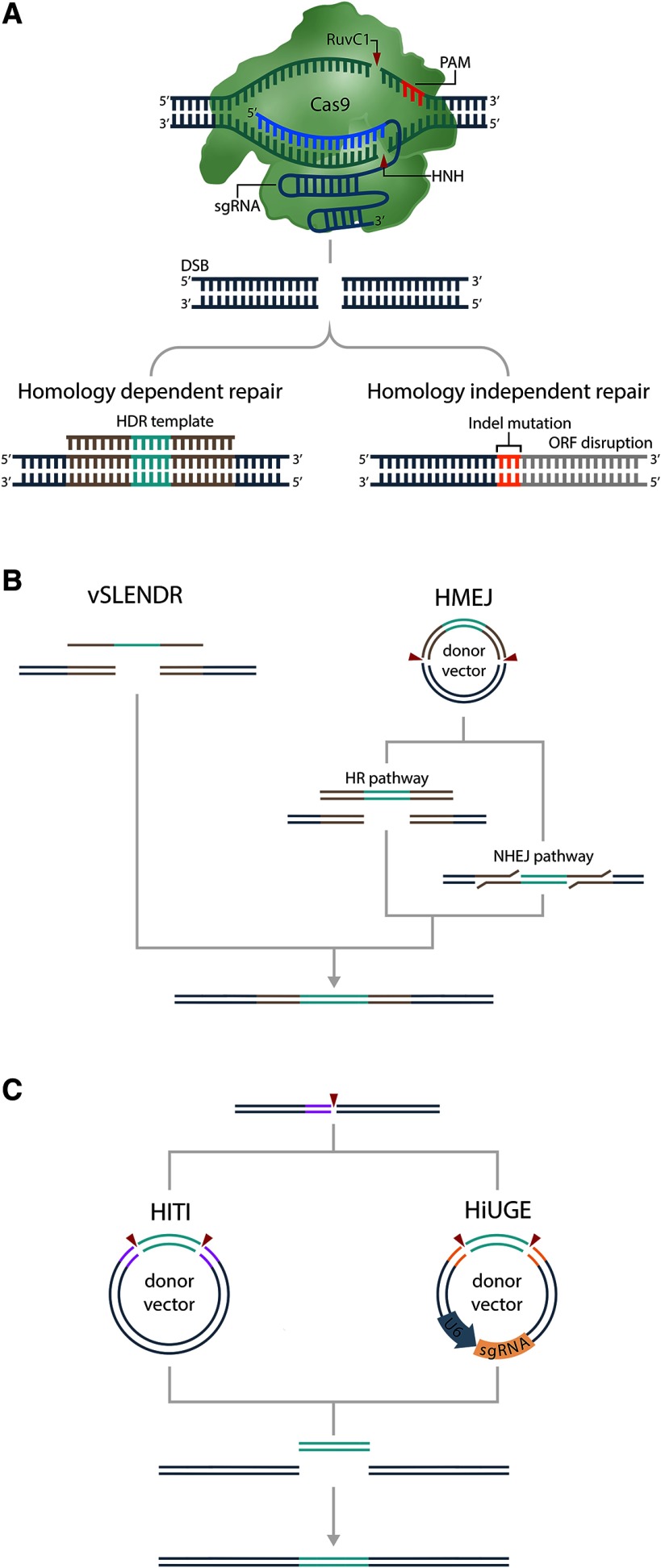
CRISPR-Cas9 mediated genome editing. ***A***, Cas9 target recognition occurs through sequence complementarity between a Cas9-associated sgRNA and a genomic target sequence. Target recognition requires the presence of a proximal 3′ PAM, which facilitates Cas9 binding and endonucleolytic cleavage. Cas9’s dual catalytic domains, HNH and RuvC1, mediate complementary and non-complementary strand cleavage, respectively. DSBs repaired by NHEJ can introduce short insertion/deletion (indel) mutations that cause frameshifts capable of disrupting protein coding sequences, causing loss of gene function. Alternatively, HDR can be used for site-specific, sequence alteration by supplying DNA templates encoding user-specified modifications. ***B***, The vSLENDR and HMEJ knock-in strategies exploit homology-dependent repair pathways to introduce foreign sequences. vSLENDR and HMEJ both require long homology arms flanking the DSB site for efficient gene insertion. However, HMEJ utilizes a hybrid NHEJ/HDR strategy which departs from the HDR-based vSLENDR strategy by also requiring DSBs to release the donor DNA template (2B – Red arrows). ***C***, Homology-independent (NHEJ) knock-in strategies mediate sequence insertion by forming DSBs at desired target sites and donor templates simultaneously. HITI utilizes a donor template that is flanked by sgRNA recognition sites that match the genomic target. Simultaneous donor/target cleavage and repair stimulate donor template insertion. HiUGE also requires simultaneous donor and target cleavage; however, HiUGE donor vectors encode both a donor template and a self-targeting sgRNA.

The prototypical CRISPR nuclease, spCas9, is an RNA-guided DNA endonuclease that relies on an RNA duplex comprised of a CRISPR RNA (crRNA) and a transactivating crRNA (tracrRNA) for its activity (Fig. 1*A*). CRISPR RNAs direct Cas9 enzymes to their intended genomic targets, whereas tracrRNAs are responsible for stimulating Cas9’s endonuclease activity and mediating pre-crRNA processing and maturation. Although discovered as two distinct RNAs in nature, it was experimentally determined that the essential elements of the tracrRNA-crRNA duplex could be combined into a chimeric sgRNA. Therefore, genome editing using this system only requires the Cas9 protein and the sgRNA. Cas9-DNA targeting occurs when the Cas9-bound sgRNA hybridizes to its target-DNA proximal to a short sequence known as the protospacer adjacent motif (PAM), which is used for target recognition. Once Cas9 binds to the genomic target site, it creates a double stranded break (DSB) approximately three bases upstream of a PAM-containing locus with sufficient crRNA complementarity. DSB formation initiates the nonhomologous end joining (NHEJ) DNA repair mechanism, which, due to the error prone nature of this repair pathway, creates insertion and deletion (indels) mutations at the DSB site (Fig. 1*A*). If the DSB occurs within the protein coding region of a gene, a loss of protein function can occur. The deletion of relevant codons or a shift in the reading frame often creates a truncated protein, collectively leading to a null allele/gene knock-out (KO; Jinek et al., 2012; Cong et al., 2013; Mali et al., 2013). Alternatively, if a donor DNA template is provided, homology-directed repair (HDR) can occur instead of NHEJ. This phenomenon can be harnessed to specifically modify the genome at precise loci (Fig. 1*B*; Cong et al., 2013; Mali et al., 2013; Wang et al., 2013). However, HDR-mediated DNA repair via existing technology remains very inefficient, and therefore, its use in non-dividing cells (i.e., neurons) *in vivo* has limited utility (Chu et al., 2015; Maruyama et al., 2015).

The Type V nuclease Cas12a (also known as Cpf1, CRISPR from *Prevotella* and *Franciscella 1*), is a related DNA targeting enzyme that departs mechanistically from Cas9 in ways that may be advantageous. For example, unlike Cas9, Cas12a processes its own CRISPR array (crRNA precursors) into mature crRNAs, independent of any ancillary enzymes and a tracrRNA. Cas12a also recognizes a different PAM sequence (Cas12a, TTTN; Cas9, NGG), generates staggered cuts and requires a much shorter guide RNA than Cas9 (∼40 nt, Cas12a; ∼100 nt, Cas9). Cas12a’s compact guide RNA architecture and self-crRNA processing ability make it well suited for multiplexed gene-targeting, particularly through the use of custom arrays encoding multiple crRNAs. Recently, these properties were optimized and harnessed for large scale gene-editing, with Campa and coworkers reporting the ability to deliver and express 20 crRNAs and Cas12a from a single vector, simultaneously (Campa et al., 2019). The continued discovery and development of new CRISPR-Cas systems with advantageous properties is highly encouraging for the future of biomedical research and therapeutic development.

## Gene Disruption in the Mammalian Brain via CRISPR-Cas and NHEJ

Targeted gene disruption is a popular approach for dissecting the functional role of many synaptic and neuronal proteins *in vivo* (Gray et al., 2011; Uezu et al., 2016). Historically, this has required conventional mutant germline engineering, which is experimentally time-consuming, can generate deleterious phenotypes, and is generally prohibitive for multigene perturbation. Gene disruption with CRISPR-Cas has been demonstrated as a promising alternative to existing gene KO strategies. Several groups have begun to apply CRISPR-Cas to disrupt genes in mature neurons *in vitro* and *in vivo*, by targeting Cas9 to specific loci and relying on NHEJ repair pathways to create indels, leading to a high rate of gene disruption (Incontro et al., 2014; Swiech et al., 2015).

The earliest studies that implemented CRISPR-Cas for neuronal gene editing *in vivo* established the lack of toxicity of prolonged Cas9 expression in neurons while also creating the first transgenic and viral platforms for their expression and delivery (Platt et al., 2014; Swiech et al., 2015). Using these transgenic mice, Platt and coworkers also demonstrated the high KO frequencies (84% biallelic, 9% monoallelic; *NeuN*) achievable in neurons transduced with AAV-sgRNAs. Swiech and coworkers sought to expand the applicability of CRISPR for broad *in vivo* use by adapting Cas9 for packaging into popular viral vectors for gene delivery into the brain (Swiech et al., 2015). The adeno-associated virus (AAV) DNA packaging limit (∼5 kb) is a major limitation for viral delivery *in vivo*, therefore packaging the Cas9 transgene (∼4 kb), sgRNA cassette and other necessary expression components into a single vector is infeasible. To circumvent this, Swiech and coworkers developed an AAV-CRISPR system that expresses spCas9 and its respective sgRNA from separate AAV vectors. Applying AAV-CRISPR to target various genes *in vitro* and *in vivo* recapitulated the substantial editing efficiency observed in transgenic Cas9 mice. For example, targeting methyl CpG binding protein 2 (*MeCP2*) in cultured neurons produced morphologic defects concurrent with MeCP2 loss of function. Furthermore, multiplexed targeting of several DNA methyltransferase genes within the dentate gyrus was capable of producing context-specific freezing deficits in mice that received contextual fear conditioning, while sparing behavioral performance in other tasks (open field test, novel object recognition, elevated plus maze).

Traditional gene editing strategies have relied heavily on engineered viral vectors for *in vivo* construct delivery (Yin et al., 2017). Although AAV and lentiviral (LV) vectors are widely used for their ability to stably express transgenes for extended periods, the potential drawbacks of viral delivery and prolonged Cas9 expression for therapeutic gene editing have received increased attention. For example, higher cellular concentrations of Cas9 have been shown to decrease specificity, presumably because off-target cleavage is the only possibility after all target sites have been destroyed (Davis et al., 2015). This observation has raised concerns for therapeutic developments that rely on viral gene transfer, which in the case of AAV-mediated gene expression, persists for several years after delivery (Nathwani et al., 2011; Wojno et al., 2013; Colella et al., 2018; Guilbaud et al., 2019). Engineered ribonucleoprotein complexes (RNP; Cas9 protein bound to a guide RNA) and Cas9-encapsulating nanoparticles have been developed as non-viral alternatives for local, transient CRISPR expression in the brain.

Staahl and coworkers introduced a cell permeable Cas9-RNP capable of transient and titratable gene disruption (Staahl et al., 2017). Cas9-RNPs were designed with repeating SV40 nuclear localization sequences (NLS), which have been previously reported to enhance cell-penetrance (Liu et al., 2015). Preassembled Cas9-RNPs were injected into the S1 primary somatosensory cortex, the V1 primary visual cortex, the dorsal striatum and the hippocampus of Ai9-tdTomato mice. Reporter activation increased in a dose-dependent manner with larger administered doses of Cas9-RNP. Furthermore, RNP injection into the dorsal striatum did not induce a significant immune response, which has been a point of concern after reports of anti-Cas9 immune responses (Chew et al., 2016).

Recently, the nanoparticle-based CRISPR-Gold system was used to target mGluR5, a metabotropic NMDA receptor involved in autism spectrum disorder (ASD)-related hyperexcitation (Lee et al., 2018). CRISPR-Gold RNPs containing mGluR5-targeting guides were infused into the striatum of fragile X mental retardation 1 (*FMR1*) KO mice, which significantly reduced exaggerated stereotypies (excessive digging and jumping). Analysis revealed 14.6% of striatal *mGluR5* genes contained loss of function (LOF) mutations, while mGluR5 mRNA and protein levels decreased by roughly 50%. Despite modest editing efficiency, these results highlight the potential of nanoparticle-based systems to deliver CRISPR and therapeutically edit genes in the brain. While CRISPR-Gold administration was sufficient to reverse the behavioral phenotype, additional optimization of nanoparticle entry into neurons will likely expand the use of non-viral, nanoparticle-based methods for genome editing in neuroscience.

Another group engineered membrane-permeable nanocomplexes to deliver Cas9 RNPs into the brain (Park et al., 2019). CRISPR nanocomplexes were generated by fusing an amphiphilic R7L10 peptide to Cas9 RNPs to permit cellular entry. R7L10-Cas9-RNPs exhibited remarkable *in vivo* stability and longevity, sustaining high levels of expression for over a week, which declined below detection thresholds after three weeks. Unlike virally delivered CRISPR transgenes that remain stably expressed for extended periods, nanocomplex-delivered RNPs possess limited opportunity to perform their gene targeting functions. Remarkably, *in vivo* targeting of β-secretase 1 (*Bace1*) in the hippocampal CA3 region of 5XFAD transgenic mice produced an editing efficiency of 45% which significantly reduced Aβ plaques and Aβ42 secretion. Surprisingly, a single hippocampal injection of *Bace1-*targeting nanocomplexes elicited persistent improvements in contextual and associative memory three months after treatment (Park et al., 2019). While the decay rates of injected RNPs and their potential off-targeting effects remain to be determined, additional research could accelerate the development of injectable RNP therapies for focal neurologic disease.

## Germline Editing with CRISPR

Genetically modified animals have been instrumental in understanding genetic contributions to neuronal development, function and disease. Conventionally, establishing transgenic animal strains has been a time- and labor-intensive process that requires several months and specialized facilities for completion (Capecchi, 2005). In recent years, many of these constraints have been overcome by CRISPR-Cas9 genome editing. The ability to rapidly produce transgenic animals harboring multiple germline mutations with relative ease is a significant improvement over traditional transgenic animal production approaches. For a more detailed discussion on generating transgenic/knock-in mice with CRISPR-Cas, we direct the reader to the following articles (Yang et al., 2014; Henao-Mejia et al., 2016; Williams et al., 2016).

While the broad availability of genetically modified mice has contributed to their widespread use in biomedical science, rats remain the preferred animal model in behavioral neuroscience research. The paucity of available transgenic rat models has left an unmet demand for additional transgenic rat lines (Ellenbroek and Youn, 2016). Germline genome editing with CRISPR-Cas9 has emerged as a highly efficient method for producing transgenic strains. As such, CRISPR-Cas9 was used to generate transgenic Cre-dependent Cas9 and Cre-dependent Cas9-nickase [Cas9(D10A)] rats and an improved Cre recombinase (iCre) rat line regulated by the dopamine transporter promoter (DAT-iCre) (Bäck et al., 2019). To show that gene targeting was Cre dependent, Back and coworkers infused AAVs encoding iCre and tyrosine hydroxylase (TH)-targeting sgRNAs into the midbrain. Four weeks after infusion, a 45% and 60% decrease in TH immunoreactivity was observed in the substantia nigra and striatum, respectively. To determine the targeting efficiency achievable with double-transgenic animals (DAT-iCre+/Cas9+), AAVs encoding *Manf* sgRNAs were infused into the midbrain. After four weeks, only 3% of dopaminergic neurons demonstrated *Manf* immunoreactivity; additionally, nearly 90% of non-dopaminergic neurons remained *Manf*^+^, thereby illustrating the potential of these lines to facilitate highly specific genome editing with extremely high editing efficiencies. With the availability of neuron-specific Cre-driver lines (GABAergic, D1, D2, parvalbumin), these Cre-dependent Cas9 rat lines present a significant advancement for future gene studies in behavioral neuroscience.

## Gene Modification in the Mammalian Brain via CRISPR-Cas and HDR

Currently the factors governing DNA repair pathway choice remain unclear. In general, NHEJ appears to be far more efficient and active compared to HDR (Cox et al., 2015). It is generally believed that HDR is mostly restricted to the S/G_2_ phases of the cell cycle, which may restrict harnessing HDR’s full potential in postmitotic cells such as neurons (Saleh-Gohari and Helleday, 2004). This may be due to phase specific conditions favorable to recombination such as the presence of proximal sister chromatids or the increased expression of requisite repair machinery. Both are conditions which may preclude robust HDR activity in terminally differentiated neurons. However, this remains to be determined.

Although Cas9’s canonical function is to cleave DNA, it can also be used to introduce foreign transgenes and new sequences using HDR (Fig. 1*B*). Low neuronal HDR activity has largely discouraged gene-editing attempts in the brain. However, recent evidence has surfaced demonstrating the successful modification of neuronal genes *in vivo*. Using their newly developed CRISPR-based HDR system dubbed SLENDR (single-cell labeling of endogenous proteins via HDR system), Mikuni and coworkers targeted neural progenitors at embryonic days (E)12 and E15, when HDR should be active. Embryonic brains were subjected to *in utero* electroporation (IUE), to deliver sgRNAs, Cas9 coding plasmids, and a hyperactive Piggyback transposase system to allow the stable integration of these transgenes and donor templates consisting of single stranded oligonucleotides (ssODNs). This approach enabled the modification of targeted genes to possess N-terminal or C-terminal epitope tags. Remarkably, SLENDR was also capable of inserting large sequences into endogenous loci, such as the GFP-coding region, facilitating protein localization studies. The authors reported modification frequencies as high as 7.5% of neurons when targeting was performed at E12, and slightly lower levels when performed at E15. It is important to note that indel formation will occur at a much higher frequency compared to HDR-mediated sequence insertion using this system. Additionally, the authors specifically targeted the beginning and end of the protein coding regions, to reduce the chance that indel formation would have a consequence on protein structure and function.

Nishiyama and coworkers adapted the SLENDR system for viral delivery. This system, referred to as vSLENDR (AAV/CRISPR-based viral-mediated single-cell labeling of endogenous proteins via HDR system), was shown to allow HDR-mediated gene modification of neurons in the mouse adult brain (Fig. 1*B*). They observed gene modification efficiencies *in vivo* (Nishiyama et al., 2017) as high as ∼30% of targeted neurons, which provides proof-of-principle for HDR-mediated editing in mature neurons. While encouraging, the mechanism of HDR-mediated editing requires additional characterization and subsequent optimization before it can be broadly applied for *in vivo* studies.

## Additional Gene Modification Strategies

While broadly considered an inherently error-prone process, various NHEJ-dependent DNA-editing tools have been developed that demonstrate the remarkably high editing frequency and precision of NHEJ repair (Fig. 1*C*). These tools, designated homology-independent (HI) targeted insertion (HITI), Homology-Mediated End Joining (HMEJ) and Homology-independent Universal Genome Engineering (HiUGE) have been shown to effectively integrate exogenous DNA sequences at similar frequencies (20% to over 50%). The first of these, HMEJ, exploits homology-dependent (HD) processes by coupling donor templates harboring sgRNA recognition sites with targeted, Cas9-mediated DNA cleavage. HMEJ-DNA donors contain 5′ and 3′ distal sgRNA sites that, upon cleavage, release a long donor cassette which encourages integration into the cleaved genomic site. When applied to adult mouse neurons *in vivo* HMEJ produced knock-in frequencies of ∼50%. Although HD strategies ensure locus specificity through extensive donor template homology, unique template production is generally restrictive for high-throughput experimentation. Therefore, unrestricted by locus homology, HI systems have gained more traction. The HITI and more recently developed HiUGE systems also exploit NHEJ repair to introduce DNA payloads. Both HITI and HiUGE incorporate similar components and mechanisms to achieve targeted transgene integration. For example, the use of a non-homologous donor vector with sgRNA recognition sequences is ubiquitous among NHEJ-mediated systems. However, HITI and HiUGE depart as HiUGE donors contain self-targeting sgRNAs, while HITI donors require sgRNA recognition sequences to be manually matched between the target and donor. The addition of a self-targeting guide RNA to HiUGE vectors permits the development of “all-in-one” donor libraries that may function complimentarily with large-scale CRISPR genetic screens.

## Regulable Gene Editing with Inducible CRISPR-Cas Systems

Germline editing with CRISPR-Cas9 has proven remarkably useful for genetically modifying animals (Li et al., 2013; Chapman et al., 2015; Remy et al., 2017). However, germline modifications can produce undesirable developmental phenotypes providing little benefit for studies interrogating gene function in adult animals. Furthermore, temporally precise manipulations may be required for studying gene function in dynamically regulated processes. In such situations it may be beneficial to deploy temporally regulable systems capable of gene editing within tightly restricted windows. Towards this aim, CRISPR-Cas9 has been combined with several other technologies to develop systems that can be regulated genetically, optically, or with small molecules (Dow et al., 2015; Zetsche et al., 2015).

**Figure 2.**
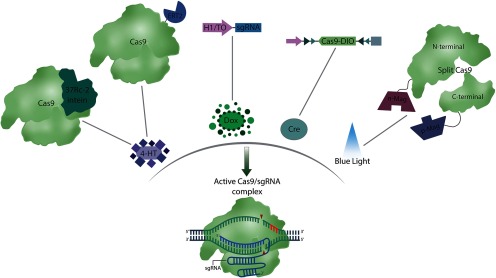
Inducible CRISPR-Cas systems. CRISPR-Cas9 genome editing can be spatially and temporally regulated with a variety of genetic, small molecule, and optical techniques. Intein-Cas9 and iCas can be regulated with the small molecule 4-hydroxytamoxinfen (4-HT). Whereas 4-HT-induced intein splicing renders Intein-Cas9 constitutively active, iCas is bidirectionally regulable. Gene targeting sgRNAs can be transcriptionally regulated with the Dox response H1/TO promoter. Additionally, both sgRNA and Cas9 expression cassettes can be rendered Cre dependent with the insertion of flanking loxP sites. Split architecture Cas9 systems have also been rendered photoinducible through fusions to light responsive, heterodimerizing molecules.

Some of the first inducible CRISPR systems were regulated by components of the popular tetracycline-dependent promoter (Tet) system (Dow et al., 2015; de Solis et al., 2016), which can be regulated in Tet-on (rtTA) and Tet-off (tTA) configurations (Fig. 2; Gossen and Bujard, 1992; Gossen et al., 1995). de Solis and coworkers developed the first doxycycline (Dox)-inducible Cas9-based editing system that saw use in the brain. First, Cas9 was placed under the control of the Dox-dependent TRE3G promoter in an to attempt to temporally regulate Cas9 expression and subsequent genome editing (de Solis et al., 2016). However, TRE3G-driven Cas9 exhibited leaky expression *in vitro,* prompting the development of regulable sgRNA expression vectors, which successfully regulated gene-editing events in a Dox-dependent manner. To determine whether this Dox-regulable CRISPR-Cas9 system was suitable for *in vivo* applications, AAV vectors encoding Cas9 and Dox-inducible sgRNAs were infused into the basolateral amygdala (BLA). *In vivo* genome-editing analysis revealed that only animals receiving Dox contained indels at the target locus. Additionally, Dox-inducible and constitutively expressed systems exhibited near identical levels of gene editing, demonstrating that spatiotemporally precise editing is achievable in the brain without significant loss of efficiency. Additional Cre and Dox-inducible CRISPR systems have been developed based on the smaller SaCas9 endonuclease. For further discussion of the SaCas9 orthologue and these inducible tools, we direct the reader to (Kumar et al., 2018; Zhou et al., 2018b).

While the conditional Tet- and Cre- based systems are frequently used to restrict gene expression temporally and spatially, their specificity and regulation is largely transcriptionally mediated. In cases where swift gene-editing is desirable, it is beneficial to reduce the response rate of the system. Post-translationally regulated processes circumvent the *de novo* transcription and translation involved in transcriptionally mediated responses, permitting a more rapid response to dynamic cellular environments. Additionally, reducing the permissible window for gene-targeting events could significantly reduce the off-target modifications reported with constitutively active Cas9. Several inducible Cas9 enzymes have been developed whose activities are post-translationally regulated with small molecules (Fig. 2; Davis et al., 2015; Liu et al., 2016a). These small molecule-responsive systems use the human estrogen receptor ligand-binding domain (ERT) fused to Cas9 to trigger gene editing events in the presence of the ERT ligand 4-hydroxytamoxifen (4-HT). Davis and coworkers introduced a 4-HT-inducible Cas9 nuclease whose enzymatic activity was inhibited by a strategically placed, self-splicing intein (Intein-Cas9; Davis et al., 2015). Intein-Cas9 was engineered such that its enzyme activity would only be restored after administration of 4-HT, which activates intein-protein self-splicing and permits Cas9’s adoption of a catalytically active form. A related 4-HT inducible Cas9 enzyme was introduced in 2016, dubbed “iCas.” However, this system departs from its predecessor by employing ERT2 as a subcellular carrier versus a covalent inhibitor. As the ERT2 ligand binding domain permits translocation into the nucleus when bound by 4-HT, fusing multiple copies of the ERT2 domain to Cas9 enables bidirectionally regulable genome editing in human cells. Both of these systems demonstrated improved editing specificities over wild-type Cas9, although iCas9 exhibited lower background activity and higher on-target editing when benchmarked against intein-Cas9. While intein-Cas9 and iCas9 show promise for studying dynamic processes in the brain, to our knowledge, they have yet to see use in such experiments.

Advances in photoinducible protein biology have culminated in the development of systems that can control gene-editing and transcription with blue-light irradiation (Fig. 2; Nihongaki et al., 2015; Polstein and Gersbach, 2015). Nihonkagi and coworkers achieved photoinducible gene editing by conjugating fragments of a Cas9 nuclease to protein elements of a dimerizing, light responsive system dubbed “magnets” (Kawano et al., 2015). The fungal-derived magnet system consists of two photoinducible protein elements termed “positive magnet” (pMag) and “negative magnet” (nMag), which are named on the basis of their electrostatic properties (Kawano et al., 2015). This system demonstrated that gene editing could be bidirectionally regulated by light irradiation, albeit with modest indel frequencies and a relatively slow response time (maximal editing ∼48 h). As these limitations may limit paCas9’s usefulness *in vivo*, additional engineering and optimization are likely required before this technology can be robustly applied in animal studies. While light inducible and optogenetic technologies are widely used in neuroscience research, photoactivatable gene-editors have yet to be applied to the nervous system.

## Genomic Regulation with Nuclease-Deficient Cas9

**Figure 3.**
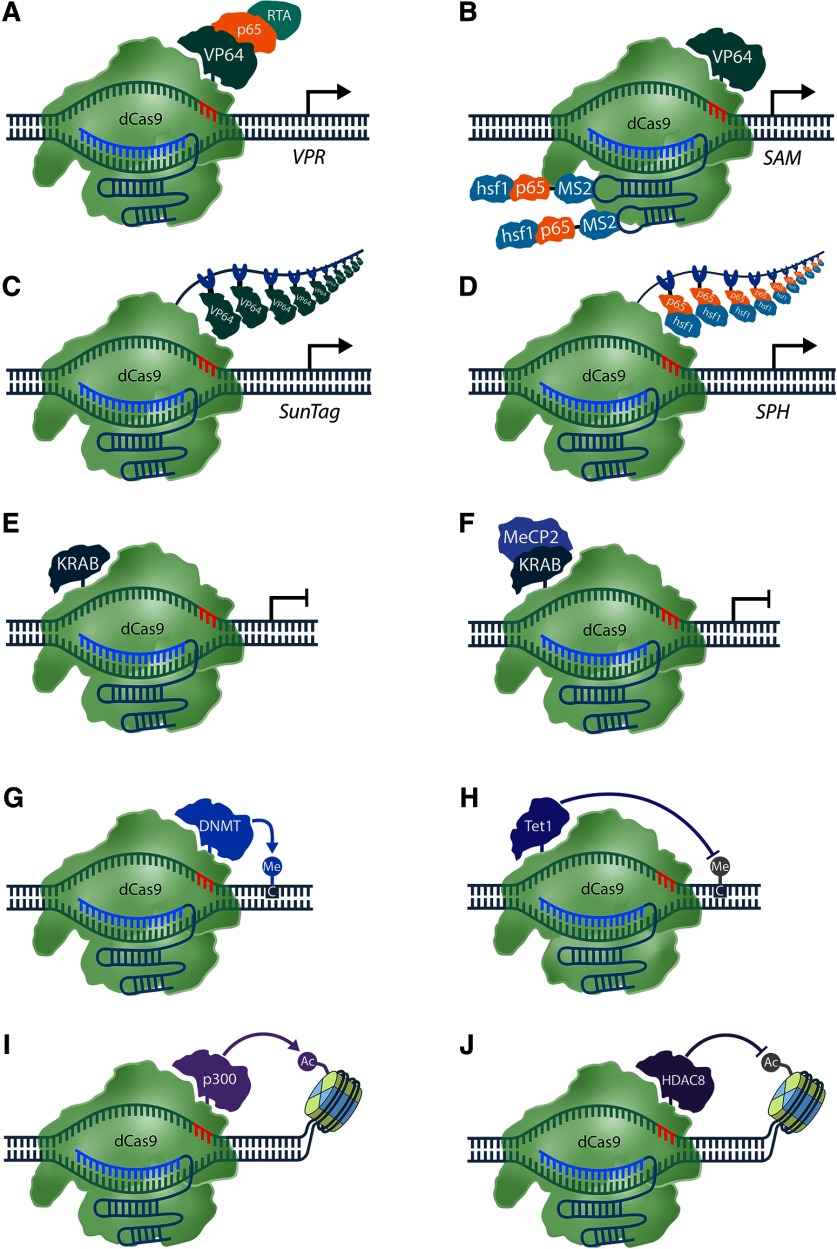
Transcriptional and epigenetic regulation with CRISPR-Cas transcriptional control can be achieved by fusing various transcription regulating enzymes to catalytically inactive Cas9 (dCas9). The CRISPR-based activators SunTag, VPR, SAM, and SPH employ various architectures to recruit transcription activating molecules. ***A***, VPR deploys traditional peptide linkers to fuse the tripartite VP64, p65, and Rta effector to dCas9. ***B***, The synergistic activator mediator (SAM) uses the MS2 RNA aptamer to recruit MCPs fused to a p65-HSF1 domain to induce transcription. ***C***, The SunTag system utilizes the a GCN4-epitope array to localize VP64 activators to transcription start sites (TSSs). ***D***, Relatedly, the SPH system uses the SunTag scaffolding array to recruit p65-HSF1 dimers in lieu of VP64. The dCas9-KRAB (***E***) and the improved dCas9-KRAB-MeCP2 (***F***) transcriptional repressors use similar strategies inhibit transcription. ***G***, dCas9 fused to the DNA methyltransferase 3A (DNMT3A) enzymatic domain can de-novo methylate CpG dinucleotides in a programmable manner. ***H***, dCas9 fused to ten-eleven translocation’s (TET1) catalytic domain facilitates successive cytosine oxidation and demethylation at methylated CpG sites. dCas9-DNMT3A/TET1 can effectively regulate gene transcription by targeting CpG containing promoter regions for epigenetic modification. dCas9 C-terminally fused to the catalytic core of the human p300 acetyltransferase (p300^core^) ***I*** or histone deacetylase 8 (HDAC8) ***J*** can regulate the acetylation status of histone 3 lysine 27 (H3K27) residues to regulate transcription from promoters and both distal and proximal enhancers.

Cas9’s capabilities have expanded beyond conventional genome editing by adapting the system into a programmable DNA-binding module (Fig. 3). To achieve this, Cas9’s catalytic activity was abolished by introducing point mutations into the RuvC1 (D10A) and HNH (H840A) domains to generate nuclease deficient or dCas9. Catalytically-inactive Cas9 retains DNA-binding capability with no apparent loss of targeting or binding specificity (Qi et al., 2013). As discussed below, dCas9-effector fusions provide seemingly endless applications for non-mutagenic genome modification, including transcriptional regulation, epigenome editing, cellular imaging, and RNA interference (RNAi).

## Transcriptional Regulation with dCas9

CRISPR-based transcriptional regulators provide researchers with the ability to assess the functional relevance of specific genes in a variety of neuronal contexts. By manipulating endogenous loci, CRISPR-based overexpression and gene silencing strategies circumvent the shortcomings of cDNA overexpression and RNAi-mediated silencing such as potential protein mislocalization or widespread off-targeting. The first systems endowing activator and repressor capabilities to the CRISPR platform used fusions of tetrameric herpes simplex viral protein 16 (VP64), the NF-κB trans-activating subunit p65 (p65), or the Krüppel-associated box domain of Kox1 (KRAB) to dCas9 (Gilbert et al., 2013; Fig. 3*E*). When directed to promoter or enhancer sequences, dCas9-VP64 and dCas9-KRAB were capable of inducing or suppressing gene-specific transcription, respectively. These capabilities encouraged their ready adoption for mapping putative *cis*-regulatory elements in neurodevelopment and neurodegeneration studies (Frank et al., 2015; Heman-Ackah et al., 2016; Huang et al., 2017). Although this first generation of transcriptional regulators could modestly alter transcription, several reports demonstrated that gene expression could be amplified with the provision of multiple sgRNAs per targeted promoter (Gilbert et al., 2013; Maeder et al., 2013; Konermann et al., 2015; Savell et al., 2019). This observation suggested that the overall copy number and enzyme cooperativity of the recruited effectors was responsible for differences in gene expression. Capitalizing on this observation, other groups developed additional CRISPR activator (CRISPRa) and CRISPR interference (CRISPRi) systems with enhanced transcriptional regulatory capabilities (Tanenbaum et al., 2014; Chavez et al., 2015; Konermann et al., 2015). These second-generation systems employ diverse scaffold architectures to recruit transcriptional regulators and maximize effector potency and recruitment.

Early second-generation systems employed an epitope-based scaffolding strategy to increase activator recruitment known as SUperNova (SunTag; Fig. 3*C*; Tanenbaum et al., 2014). The SunTag scaffold is a peptide array composed of tandem repeating GCN4 epitopes. Transcriptional regulators conjugated to short-chain variable fragments (scFv) with high affinity for the GCN4 epitope can effectively bind the SunTag scaffold, facilitating the formation of multimeric regulatory structures at targeted DNA sequences. Essentially the system is designed to recruit many VP64 transcriptional activation domains to the promoter to enhance transcriptional activation. Indeed, expressing dCas9-SunTag with scFv-bound VP64 activators dramatically increased targeted gene expression compared to that achieved by dCas9-VP64.

Another study (Konermann et al., 2015) examined the regulatory potential of sgRNAs designed to recruit transcriptional activators using RNA aptamers (Fig. 3*B*). Analysis of sgRNA secondary structures identified regions that were non-interacting with the Cas9 endonuclease and found that mutating distal base pairs in these regions had no influence on DNA binding or cleavage. By substituting sgRNA stem loops with MS2 aptamers that could recruit MS2 coat proteins (MCPs) fused to p65 and heat shock factor 1 (HSF1), it was determined that dCas9-VP64 could upregulate transcription at significantly higher levels when co-expressed with RNA aptamer-containing sgRNAs versus standard sgRNAs.

A separate group screened putative activator domains for gene activation potency, identifying VP64, p65 and the Epstein–Barr virus R transactivator (Rta) as the most potent transcriptional activators. However, dCas9-p65 and dCas9-Rta both exhibited lower transcription rates than the original dCas9-VP64 chimera. To overcome this, combinations of activators were fused with the aim of cooperatively inducing higher gene expression (Chavez et al., 2015). Using dCas9-VP64 as a starting framework, a tripartite fusion of VP64-p65-Rta (VPR; Fig. 3*A*) was tethered to dCas9 and subsequently assayed for induction capacity, which revealed that gene expression was upregulated between 22- and 320-fold when compared with dCas9-VP64.

Second-generation activators were screened for maximal induction of *Ascl1* and *Neurod1* genes in HEK293T cells, revealing SAM (Konermann et al., 2015), SunTag (Tanenbaum et al., 2014), and VPR (Chavez et al., 2015) as the most potent gene activators. Subsequent assays revealed SAM as the most consistent in activating high levels of gene expression. Notably, the increased transcription of several tested genes reached orders of magnitude above that induced by dCas9-VP64 (Chavez et al., 2016).

While newly developed CRISPRa systems undergo validation in several common cell types, few have seen any use in neuronal contexts. Savell and coworkers have recently introduced LV vectors capable of robust neuronal VPR expression *in vitro* and *in vivo (in vivo* discussion continued below; Savell et al., 2019). Gene overexpression assays in primary cultured neurons demonstrated VPR’s ability to robustly overexpress single or multiple genes with high specificity. Notably, multiplexed gene activation with VPR recapitulated earlier reports of sgRNA-dose responsiveness. Effective activation was demonstrated using an individual sgRNA and significantly increased with the provision of multiple unique sgRNAs.

In contrast to transcriptional activators, few dCas9 repressors capable of enhanced transcriptional downregulation have been developed. Recognizing this deficit, Yeo and coworkers proceeded to perform a similar screen to identify dCas9-repressors capable of robustly inhibiting gene expression (Yeo et al., 2018). Of the screened transcriptional repressors, the bipartite dCas9-KRAB-MeCP2 fusion emerged as the most potent (Fig. 3*F*).

## Regulating Transcription *In Vivo* with dCas9

Ectopic gene overexpression mediated through viral vector delivery is a popular strategy to investigate neuronal gene regulation (Lentz et al., 2012; Haggerty et al., 2020). As previously described, numerous CRISPRa systems have been developed, enabling the potent activation of multiple genes in various tissues types. However, until recently, these technologies have been limited to *in vitro* applications because of the difficulty associated with the efficient delivery of multiple large transgenes *in vivo*. Recently, elements of the SAM and SunTag system were combined to develop a new dCas9-based transcriptional activator, dCas9-SunTag-p65-HSF1 (SPH; Fig. 3*D*), for *in vivo* gene regulation (Zhou et al., 2018a). To develop the SPH platform, the VP64 tetramers in the SunTag system were replaced with the p65-HSF1 domains from the SAM system. When combined, these components potently induced gene expression, surpassing the SunTag, VPR, and SAM activators.

In order to circumvent the difficulties associated with viral delivery of large, multicomponent systems to the nervous system, the authors generated a transgenic mouse line harboring a Cre-dependent SPH system. Considering that cell-type and circuit-specific multiplex strategies will likely be required to successfully interrogate gene networks *in* vivo, Zhou and coworkers performed feasibility experiments on SPH’s multiplex gene activation capabilities. Using a combination of AAV vectors encoding Cre recombinase (hSyn-Cre and CamKIIα-Cre) and sgRNA arrays targeting multiple genes (eight coding genes and two long noncoding RNAs), Zhou and coworkers successfully overexpressed several targeted genes simultaneously. When coupled with existing genome wide CRISPRa sgRNA libraries, these SPH mice provide a useful tool for endogenous gene overexpression and genome wide screening in the brain.

Savell and colleagues sought to optimize the previously developed dCas9-VPR activator for behavioral neuroscience by developing neuron-optimized viral vectors capable of potent, multiplexed gene expression *in vivo*. By examining VPR expression under the control of several promoters, they were able to identify and produce a LV system that permitted robust VPR expression *in vitro* and *in vivo* under the control of the neuron-specific Synapsin promoter. This neuron-optimized LV VPR system was applied in various neuronal contexts and was notably capable of potent, isoform-specific induction of various BDNF transcripts *in vivo* (Savell et al., 2019).

Until recently, RNAi and conditional Cre-loxP systems have been the predominate methods used for gene knock-down and KO, respectively. However, evidence documenting the significant off-target effects of short hairpin RNA (shRNA) and small interfering RNA (siRNA) has accumulated (Castanotto and Rossi, 2009; Jackson and Linsley, 2010; de Solis et al., 2015). Alternative methods for gene knock-down such as CRISPR-based repressors have been proposed, due to their ability to potently silence gene expression within various contexts. However, the application of CRISPRi technology in neurons has seen limited use *in vivo*.

Recently, a LV-based CRISPRi system was developed for use in the mammalian brain (Fig. 3*E*). Using the dCas9-KRAB repressor, synaptotagmin I (*Syt1*), vesicle associated membrane protein I (*Vamp1*), syntaxin 1A (*Stx1a*), and synaptosome associated protein 25 (*Snap25*), genes responsible for vesicular neurotransmitter release, were targeted in cultured hippocampal neurons. To compare the efficiency of CRISPRi and RNAi-mediated knock-down, sgRNAs and shRNAs were tested for each target gene. CRISPRi produced ∼90% reduction in mRNA and protein levels of all genes targeted, compared to a modest reduction produced by RNAi. Additionally, whole-cell patch-clamp recordings of CRISPRi-targeted hippocampal neurons revealed significant reductions in EPSCs, as expected from disruption of the neurotransmitter release pathway (Zheng et al., 2018).

Numerous studies have reported the potential for Cas9 endonucleases to bind off-target sites (Kuscu et al., 2014; Wu et al., 2014). This, coupled with the observed potency of the dCas9-KRAB repressor, raises concerns for severe off-target silencing. The authors used a “pseudo-target fishing strategy” to determine the frequency of off-targets by expressing dCas9-KRAB with sgRNAs containing unique mismatches with the *Syt1* locus. This strategy revealed that *Syt1* expression levels remain largely unchanged, indicating that mismatched sgRNAs were incapable of efficiently directing dCas9-KRAB to the *Syt1* locus (Zheng et al., 2018).

As cell-type specificity is essential for the interrogation of gene and cell function in the brain, the dCas9-KRAB repression system was modified to restrict targeting to glutamatergic (CaMKIIα-dCas9-KRAB) or GABAergic (VGAT-dCas9-KRAB) neurons. LV infusion into the dentate gyrus revealed a roughly 20% transduction rate of neurons confined to the granule layer. Analysis of dCas9-KRAB^+^ DG neurons revealed that *Syt1* expression was completely abolished in a cell-type specific manner. Likewise, whole-cell patch-clamp revealed that EPSCs within CaMKIIα-expressing neurons were almost completely abolished, with a similar reduction in GABAergic neuron IPSCs (Zheng et al., 2018).

Targeting *Syt1* within glutamatergic and GABAergic neurons enables altering of the inhibitory-excitatory (I-E) ratio within the hippocampus. As the hippocampus is implicated in various forms of learning and memory (LaBar and Cabeza, 2006), the authors subjected mice to multiple spatial and associative learning tasks after CRISPRi mediated I-E shifting. Animals receiving CaMKIIα-dCas9-KRAB (shift towards inhibition) exhibited significant performance reductions in spatial memory related tasks (Morris water maze, Barnes Maze, T maze) compared with animals receiving VGAT-dCas9-KRAB (shift towards excitation). In tests of associative memory (fear conditioning), CaMKIIα driving animals demonstrated reduced freezing levels in response to a cued stimulus (tone) in contrast to VGAT driving animals which exhibited slightly enhanced freezing, illustrating that alterations of the I-E ratio within the hippocampus could bidirectionally regulate spatial and contextual fear memory (Zheng et al., 2018).

## CRISPR-Based Epigenome Editors

DNA methylation is vitally involved in neurodevelopment and in dynamic gene regulation across various networks in the CNS (Smith and Meissner, 2013). Cytosine methylation within promoter regions permits the controlled regulation of various processes ranging from basic gene transcription to higher-order functions such as learning, memory and cognition. Historically, epigenetic studies have been incapable of determining the functional relevance of specific methylation events due to the limitations of the methylation-inhibiting small molecules 5-azacytidine and 5-aza-2′-deoxycytidine (Heerboth et al., 2014). Although these compounds could be locally injected to induce regional CpG hypomethylation, these shortcomings are largely prohibitive for the precise investigation of disorders such as Angelman’s, Fragile X, Rett syndrome, and Prader–Willi syndrome, all which exhibit significant neurologic phenotypes and aberrant CpG methylation (Butler, 2009). Recent advances in epigenome engineering technology have produced CRISPR-based epigenome editors that couple the programmable targeting of CRISPR with enzymes involved in the DNA methylation pathway (Fig. 3*G–J*; Liu et al., 2016b, 2018a; Lei et al., 2017).

As dynamic DNA methylation has been proposed to regulate activity-dependent gene expression, Liu and coworkers sought to determine whether their LV dCas9-TET1 system could induce brain-derived neurotrophic factor (BDNF) expression by targeting the BDNF IV promoter for demethylation in cultured primary neurons (Fig. 3*H*; Liu et al., 2016b). Neuronal dCas9-TET1 expression successfully increased BDNF expression 6-fold. However, “no sgRNA” controls also produced a nearly 2-fold increase in BDNF expression, demonstrating this system’s potential for non-specific gene induction. CRISPR-epigenome editors have also been used preclinically for therapeutic studies. For example, dCas9-TET1 was used to demethylate the CGG trinucleotide expansion in the 5′ UTR of the *Fmr1* gene in models of fragile X syndrome (FXS; Persico and Napolioni, 2013; Liu et al., 2018a). dCas9-TET1 targeting to the *Fmr1* 5′ UTR in *in vitro*-derived FXS neurons significantly reduced CGG trinucleotide hypermethylation and the associated hyperexcitable phenotype. Remarkably, dCas9-TET1-treated inducible pluripotent stem cell (iPSC)-induced FXS neurons retained high levels of FMRP expression months after their engraftment into live mouse brains.

Beyond the transcriptional regulation mediated by dynamic DNA methylation, histone modifications gatekeep gene expression by altering chromatin conformation and the accessibility of *cis*-regulatory elements to DNA binding proteins (Yarrington et al., 2018). CRISPR-based epigenome editors have been used to uncover the functional importance of discrete regulatory elements (Hilton et al., 2015; Chen et al., 2019). Using dCas9-p300 and dCas9-HDAC8 (Fig. 3*I*,*J*), the histone modifications at the second enhancer (Enh2) of the neuronal immediate early gene (IEG) *Fos* were shown to fine tune the degree of activity-induced transcription. In other words, the type of histone modification installed by p300/HDAC8 could slightly increase or decrease activity-dependent *Fos* transcription. However, inducing a heterochromatic state with HDAC8 could not completely silence *Fos* activity, and inducing a euchromatic or “protranscriptional” state was insufficient to induce *Fos* transcription without neuronal activity. This observation contrasts the constitutive gene activation mediated by other CRISPRa systems, which if targeted to Enh2, would presumably induce *Fos* without neuronal activity. The effectiveness of CRISPR-based epigenome editors highlights the potential for these new tools to elucidate the functional relevance of non-coding and epigenetically regulated elements to animal behavior, neuronal function and disease.

## Engineering the Neuronal Transcriptome with RNA-Targeting CRISPR Effectors

**Figure 4.**
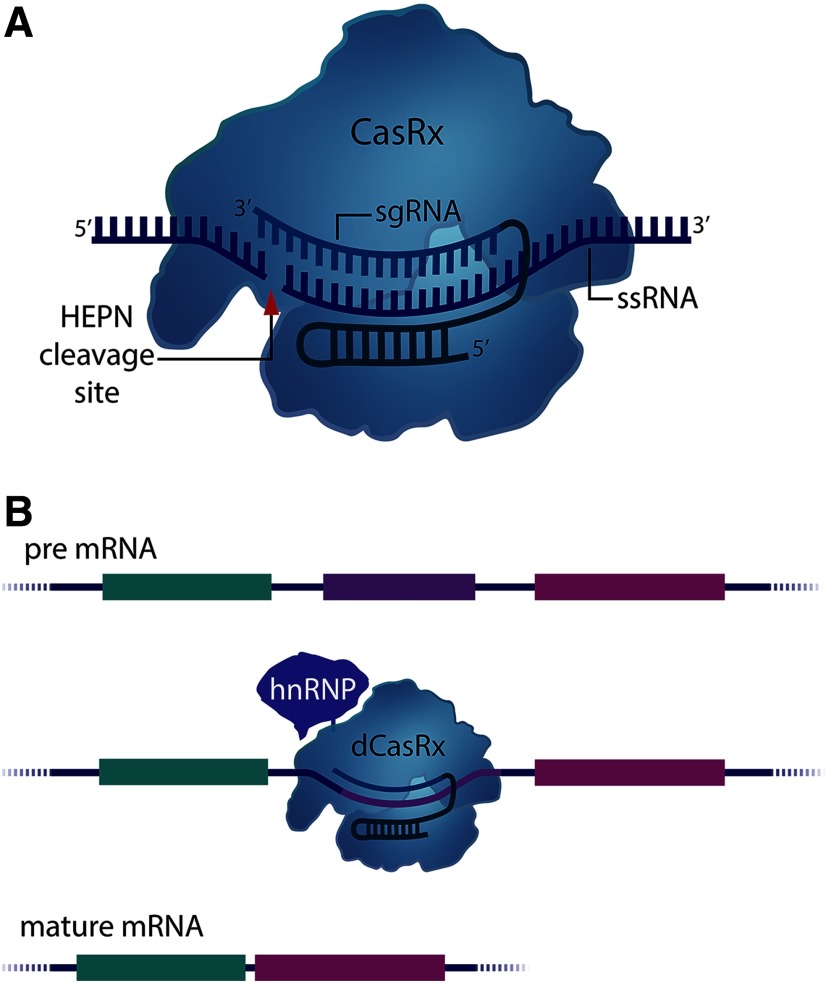
RNA targeting with CasRx. (***A***) CasRx can efficiently target and cleave RNA via its dual HEPN nuclease domains. Unlike DNA targeting Cas9 endonucleases, several Cas13 orthologues do not exhibit PFS (PAM site analogue) requirements. Mutating HEPN catalytic residues (R295A, H300A, R849A, H854A) preserves CasRx’s RNA binding ability, allowing CasRx to be adapted for fusion constructs. (***B***) Splice isoform engineering|decatalyzed CasRx (dCasRx or dCas13d) fused to the splicing factor hnRNP1 can be targeted to various splice elements (splice acceptors, splice donors, intronic branch points, etc.) to induce exon skipping and isoform selection.

**Figure 5.**
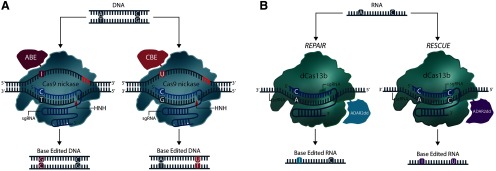
Base editors. ***A***, Adenine base editor (ABE) and cytosine base editor (CBE) catalyze the deamination and alteration of DNA nucleobases via chimeric Cas9n-DNA deaminase fusions. Nicking (single strand DNA cleavage) of the non-edited strand increases base-editing efficiency by inducing cells to repair the cleaved strand using the edited strand as a template. ***B***, The Cas13-based RNA base editor RNA-editing for programmable A to I replacement (REPAIR) mediates the conversion of adenosine to inosine, while RNA editing for specific C to U exchange (RESCUE) mediates the conversion of cytosine to uracil.

Programmable DNA-targeting Cas9 nucleases have been used for *in vivo* gene studies. However, tools enabling the study of RNA function are severely lacking. Recently, the diverse group of Class 2 CRISPR-Cas systems has been expanded to include the Type VI, RNA-targeting Cas13 family of effectors (Abudayyeh et al., 2016, 2017). Despite their recency, RNA-targeting CRISPR systems have been engineered for targetable RNA visualization, knock-down, base-editing, and *in vitro* isoform manipulation (Figs. 4, 5; Abudayyeh et al., 2017; Cox et al., 2017; Konermann et al., 2018). The Cas13 family of endonucleases are characterized by a single-effector protein containing two higher eukaryotes and prokaryotes nucleotide-binding (HEPN) ribonuclease (RNAse) domains (Abudayyeh et al., 2016). Unlike their DNA-targeting counterparts, Cas13 effectors do not require tracrRNAs for pre-crRNA processing, nor do they require PAM sequences for nucleic acid targeting and non-self-recognition. Instead, sequences that are enriched proximally to protospacer targeting sites are referred to as protospacer flanking sequences (PFSs). Notably, several Cas13 variants have been shown to not require a PFS for RNA cleavage (Cox et al., 2017). Multiple studies have reported a large amount of divergence amongst the Type VI family, often reporting little sequence conservation among Cas13 nucleases other than the characteristic HEPN RNase domains; for a more complete discussion of their individual properties we suggest reviewing (Shmakov et al., 2017).

Numerous studies have compared the knock-down ability of multiple Cas13 subtypes and orthologues to RNAi, which have overwhelmingly demonstrated that Cas13’s RNA knock-down capabilities are superior to those of shRNAs (Abudayyeh et al., 2017; Cox et al., 2017; Konermann et al., 2018). Additionally, the recently discovered Cas13d effector, *Ruminococcus flavefaciens*-Cas13d (CasRx; Fig. 4*A*), has been shown to more effectively silence gene expression than other well-established methods such as CRISPRi (Konermann et al., 2018). When targeted to the endogenous *B4GALNT1*, *ANXA4*, and *HOTTIP* genes in HEK293FT cells, CasRx demonstrated a remarkable median knock-down efficiency of 96% compared with 53% knock-down produced with sequence-matched shRNAs. Furthermore, CasRx did not generate any detectable off-target transcriptional changes, which starkly contrasts shRNA-induced silencing of an excess of 500–900 off-target genes (Konermann et al., 2018). CasRx also outperformed CRISPRi (dCas9-KRAB) mediated repression, which produced a median 53% knock-down rate when targeted to the same genes. Other recently described Cas13 subtypes have been shown to robustly knock-down RNA in mammalian cells. Compared to Cas13a (LwaCas13a-msfGFP-NLS; Abudayyeh et al., 2017) and Cas13b (PspCas13b-NES; Cox et al., 2017), CasRx demonstrated greater transcript knock-down ability (median knock-down rates; Cas13a, 80%; Cas13b, 66%; CasRx, 97%). Remarkably, of 14 sgRNAs targeted to both coding and non-coding RNA, CasRx yielded at least ∼80% transcript knock-down, suggesting that CasRx could be used to regulate any RNA in the cell.

Several degenerative diseases have been linked to mutations within individual pre-mRNA elements. For instance, mutations within exons 45–55 or exon 23 of the Dystrophin gene produce the muscle degeneration associated with Duchenne muscular dystrophy (DMD; Ousterout et al., 2015; Long et al., 2016). Likewise, neurodegenerative tauopathies such as Frontotemporal dementia with parkinsonism linked to chromosome 17 (FTDP-17) is associated with point mutations in exon 10 of the *MAPT* gene, which determines which Tau protein isoform is expressed in neurons (Boeve and Hutton, 2008). As previous studies have reported success in models of DMD using exon-skipping strategies (Nelson et al., 2019), Konermann and coworkers tested whether dCasRx could efficiently drive isoform selection by developing a dCasRx-RNA splice effector fusion (Fig. 4*B*).

Pre-mRNA splicing is mediated by interactions between *cis*-acting elements (splice acceptor/donor sites, intronic branchpoint nucleotides, etc.) and the trans-acting spliceosome (Matera and Wang, 2014). Within the cohort of pre-mRNA interacting molecules are the heterogeneous nuclear RNPs (hnRNPs), a ubiquitously expressed group of splice factors that facilitate alternative splicing by inhibiting exon exclusion (Wang et al., 2015). The hnRNPa1-CTD was fused to dCasRx and targeted to several putative splicing elements, which successfully induced exon-skipping in a fluorescence splicing reporter. In order to determine whether skipping exon 10 of the *MAPT* gene could decrease the accumulation of pathogenic (isoform 4R) tau, cortical neurons differentiated from patient-derived iPSCs were transduced with AAV encoding dCasRx-hnRNPa1 and three exon 10 targeting sgRNAs. dCasRx-hnRNPa1 mediated exon-skipping was shown to reduce 4R/3R ratios by 50%, a level similar to unaffected controls (Konermann et al., 2018).

These results demonstrate the ability of Type VI, RNA-targeting Cas13 effectors for enhanced RNAi and manipulation. In the past, applications of dCas13 effector fusions have been limited by their large size. Therefore, CasRx’s short coding sequence (∼2.9 kb) makes it highly suited for use in AAV vectors. As described above, the CasRx fusion and three sgRNAs fell below AAV packaging limitations, a characteristic that may inspire the future development of CasRx-based effectors that are capable of elucidating RNA function in the brain.

## Base and Prime Editing

**Figure 6.**
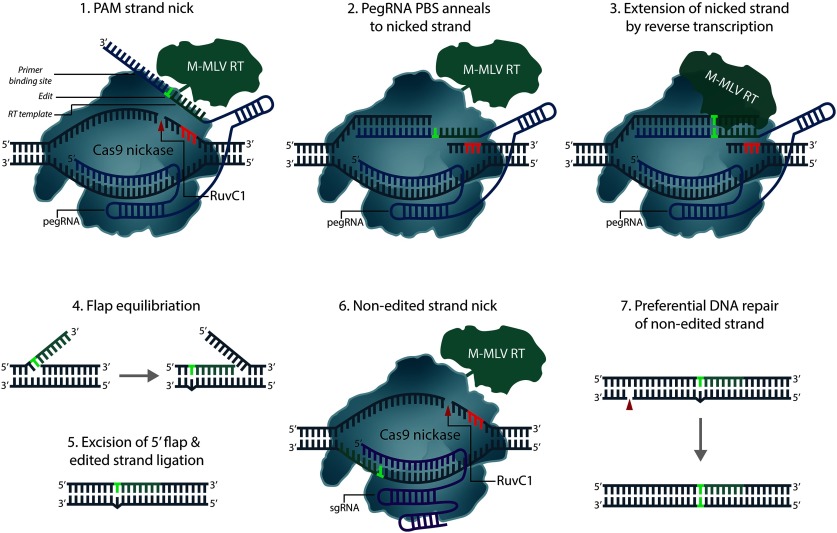
Prime editing. Prime editors (PEs) use a partially decatalyzed Cas9(H840A) nickase, a prime-editing RNA (pegRNA) and an engineered reverse transcriptase (RT) to precisely introduce DNA edits; pegRNAs contain a primer binding site (PBS) which anneals to the nicked target strand, allowing sequence extension through reverse transcription and production of the edited strand. pegRNA-PBS reverse transcription produces an edit-containing 3′ flap and an unedited 5′ flap which undergoes preferential degradation by endogenous 5’−3′ exonucleases. The remaining edited 3′ flap anneals and is ligated, resulting in a mismatched heteroduplex which can be resolved by cellular DNA repair pathways. Targeting the unedited strand with a separate sgRNA increases editing efficiency and stimulates preferential DNA repair to permanently install edited DNA.

Existing CRISPR technologies equip researchers with a powerful, multifunctional platform to investigate a staggering number of biological questions, however these tools are not without drawbacks. DSBs created by Cas9 nucleases often result in haphazard DNA repair and indel formation, which can frequently produce extensive sequence heterogeneity and yield several unwanted or deleterious DNA products. Technologies have been developed that circumvent problematic DSBs and imprecise cellular DNA repair processes through the use of enzymes (Fig. 5) that can alter RNA and DNA nucleotides *in situ*, or more recently, prime editors that can faithfully install edits through reverse-transcription of an RNA template (Fig. 6). These technologies, termed base editors, rely on dCas9 fusions to nucleobase deaminases to directly install point mutations without the need for DSBs. Existing base editors are collectively able to catalyze all possible transition mutations (C to T and A to G, point mutations) in DNA, with recent developments in RNA base editing allowing the conversion of A to I, and C to U bases as well. DNA and RNA base editors are extensively discussed in Rees and Liu (2018). As of yet, no studies have reported the use of base editors in any neuronal context. However, the growing number of single nucleotide polymorphisms (SNPs) implicated in psychiatric and neurologic diseases and the finding that the mRNAs of various neuronal ion channels and synaptic receptors undergo RNA editing may prompt the future use of these tools in neuroscience laboratories (Behm and Öhman, 2016).

Prime editors present the latest advance in precision gene editing. Anzalone and coworkers introduced a Cas9-nickase (Cas9n)-based system that couples the DSB-free editing strategy pioneered with base-editors to an sgRNA-based RNA donor template (Fig. 6; Anzalone et al., 2019), a strategy similar to one recently introduced in yeast (Sharon et al., 2018). Prime editors are multicomponent systems comprised of a chimeric Cas9n-reverse transcriptase and a prime editing guide RNA (pegRNA). Both the target locus and the desired DNA edit are encoded on the pegRNA, which harbors the standard Cas9 sgRNA components and a 3′ extended RNA template. Cas9n cleavage of the PAM-containing strand allows donor-template invasion and hybridization, which permits RNA-template reverse transcription and effective installation of the desired edit. This prime editing strategy was shown to successfully introduce broad classes of edits with lower indel frequencies than Cas9-mediated HDR in multiple cell types *in vitro*, including a modest editing frequency (6–8%) in primary neuronal cultures. Although a promising development, the frequency of genome-wide off targets and unintended reverse transcription products remain unknown. This, in concert with the modest editing frequency achieved with the latest prime editor, may preclude its immediate use *in vivo*. Nonetheless, this technology presents an exciting new development towards achieving high-fidelity, corrective gene editing with CRISPR.

## CRISPR Screens

**Figure 7.**
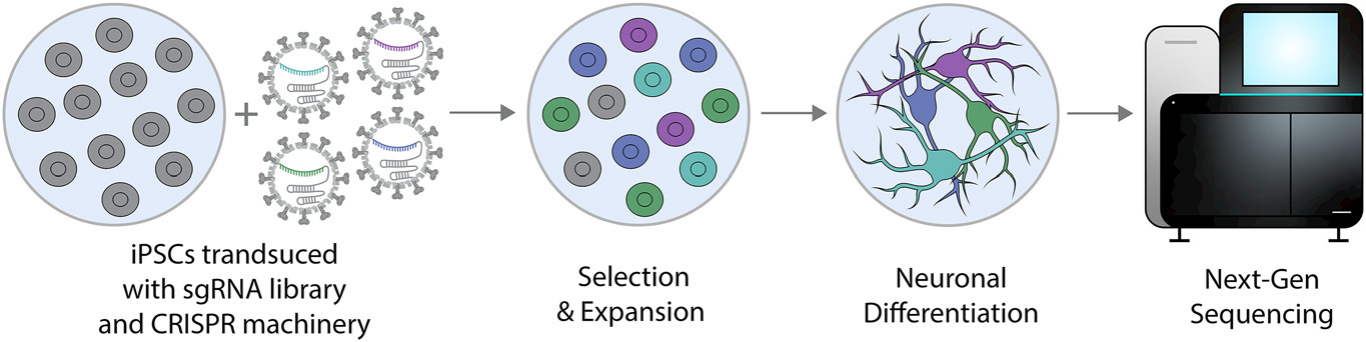
High-throughput genetic screening with CRISPR. Large scale genetic screens can be performed in iPSCs expressing CRISPR machinery. Transduction of iPSCs with pooled LV sgRNA libraries permits the selection and expansion of construct-positive cells before *in vitro* neuronal differentiation. CRISPR-KO, CRISPRi, and CRISPRa can be coupled with single cell and NGS technologies for genome-wide or targeted gain- and loss-of function screens.

The recent exponential advances in NGS technologies and the easy design and production of large numbers of unique sgRNAs has facilitated the high-throughput investigation of various psychiatric and neurodegenerative disorders, cancer, and essential gene functions through large-scale CRISPR screens (Fig. 7). CRISPR-mediated screens combine high-throughput, single-cell sequencing technologies with genome-wide sgRNA targeting libraries optimized for gene KO (Sanjana et al., 2014; Doench et al., 2016; Morgens et al., 2017; Wang et al., 2018; Liu et al., 2019), activation (Horlbeck et al., 2016; Joung et al., 2017; Chong et al., 2018; Liu et al., 2018b; Sanson et al., 2018), and silencing (Horlbeck et al., 2016; Liu et al., 2017; Sanson et al., 2018) applications. Recent applications of CRISPR-screening have produced new experimental pipelines that permit the unambiguous contribution of risk-associated genes to disease phenotypes (Thyme et al., 2019) and the determination of cellular-lineage and heredity in developmental studies (McKenna et al., 2016; Raj et al., 2018). For example, CRISPR-Cas9 was recently used to perform a mutant-phenotyping screen on schizophrenia-associated genes identified in human genome-wide association studies (GWAS; Thyme et al., 2019). Cas9 was used to mutagenize several risk-associated genes in developing zebrafish. These mutants were then subjected to behavioral and structural analysis which allowed Thyme and coworkers to successfully uncover phenotypes for multiple understudied genes. A separate zebrafish study deployed a large-scale CRISPR-Cas9 technique (GESTALT; see McKenna et al., 2016 for additional background) combined with single-cell RNA-seq (scRNA-Seq) to determine cellular fate and lineage characteristics in developing brains. Paired with an inducible Cas9, DNA barcodes harboring specific target sequences were used to indicate whether DNA editing occurred in a specific cell; because genomic bar code expression results in cellular progeny with identical barcode sequences, this allowed Raj and coworkers to determine the lineage histories for a plethora of cell types in the developing zebrafish brain.

While most screens are performed *in vitro* or *ex vivo,* two CRISPR-Cas9 mediated *in vivo* screens have recently been reported in the brain (Chow et al., 2017; Jin et al., 2019). A recent *in vivo* screen (Perturb-Seq, first described by Dixit et al., 2016) aimed at systematically uncovering the phenotypes of a large panel of ASD-related genes was performed by coupling cell-type specific transcriptomics and a lentivirus-mediated sgRNA library targeting 35 putative ASD-risk genes. Ventricularly injecting LV-sgRNA libraries into developing embryos *in utero* permitted postnatal, single-cell transcriptional profiling and identification of multiple gene clusters from cortical and striatal tissue. CRISPR knock out coupled with a scRNA-seq readout readily enabled differential gene identification, subsequent perturbation and phenotyping for a number of ASD-risk genes involved in distinct molecular pathways across variant cell types.

The earliest reported *in vivo* screen was directed at investigating the functional and tumorigenic consequences of significantly mutated genes (SMGs) that were previously identified in tumor samples taken from human glioblastoma multiforme (GBM) patients (Chow et al., 2017). A pooled AAV-sgRNA library [mouse homolog tumor suppressor library (mSTG)] targeting various risk-genes was hippocampally or ventricularly infused into mice, which produced GBM-characteristic tumor growth at four months postinjection. Histologic, transcriptomic and genetic characterization of AAV-CRISPR-mediated GBM tumors, *in vivo* and *ex vivo,* permitted the successful identification and correlation of single and co-occurring tumor drivers to GBM mutations identified in human patients.

Although CRISPR-based screens are heavily used in oncology research (Hart et al., 2015; Tzelepis et al., 2016; Chow et al., 2017), these tools have garnered significantly less attention for large-scale genetic studies in disease-relevant cell types such as differentiated neurons. Tian and coworkers recently performed several CRISPRi screens to elucidate functional contributions of various genes to cell survival, differentiation, transcriptional regulation and morphology in human iPSC (hiPSC)-derived neurons (Tian et al., 2019). In an initial survival screen, dCas9-BFP-KRAB and the LV H1 sgRNA library were used to target ∼2300 genes comprising the “druggable genome.” CRISPRi-mediated gene knock-down uncovered a strong neuronal dependence on sterol/cholesterol metabolism genes and enhanced neuron survival when members of the integrated stress response (DLK, JNK, PERK) were knocked down. Tian and coworkers also performed screens that identified common regulators of variant transcriptional programs in iPSCs and neurons, as well as several genes that contributed to neuronal longevity and morphology.

## Existing Challenges for CRISPR Gene Editing

Despite the explosive progress of CRISPR-mediated genome engineering in the last decade, significant challenges for clinical and preclinical applications remain. For example, concerns regarding CRISPR’s immunogenicity, targeting efficiency, fidelity and optimal delivery will need to be addressed before CRISPR can fulfil its clinical and research potential.

CRISPR delivery *in vivo* has been accomplished virally, with RNPs and nanoparticles. For preclinical studies using small animals, these delivery methods are sufficient, since experiments can be conducted where useful data can be generated by targeting relatively small body areas. Viruses and RNPs can deliver their necessary cargo to regions of this size reasonably well. However, improvements can be made to increase the ease of delivery and the area of tissue that could be effectively targeted with CRISPR systems. For example, conventional AAVs and LVs need to be stereotaxically injected intracranially to gain access to the brain and usually will not transduce more than a few cubic millimeters of tissue. More recently, AAVs with modified capsids have been developed that can cross the blood brain barrier, so they can be administered systemically and transduce brain cells (Chan et al., 2017). Although these developments are encouraging, they still need more development for clinical utility. Notably, non-human primate research and clinical human studies will generally benefit from less invasive routes of delivery that can target larger regions of the brain. This will be an important hurdle to overcome if CRISPR is to ever realize its full potential of treating CNS diseases.

For any gene modification technique, its specificity and accuracy are paramount, especially for clinical applications. While high-fidelity SpCas9 variants have been developed (Kleinstiver et al., 2016; Slaymaker et al., 2016; Chen et al., 2017; Casini et al., 2018; Chatterjee et al., 2018; Hu et al., 2018), the off-targeting frequencies and loci for therapeutic sgRNAs will need to be thoroughly characterized *in vitro* before use in human therapies. For gene KO in preclinical applications, SpCas9’s fidelity is likely sufficient, especially since researchers can perform independent experiments with differing sgRNAs designed to KO out their intended gene. Given that differing sgRNAs would likely not exhibit the same off targets, if the same phenotype is obtained with both sgRNAs, then their result would likely be due to the KO of the intended target.

Preclinical studies using CRISPR-Cas have generated significant enthusiasm for the future of personalized gene therapies. However, as CRISPR becomes implemented clinically, aspects of its safety for use in human therapies have received extensive scrutiny. Recently, various preclinical studies have described the immunogenicity of CRISPR nucleases following systemic (IV) administration to laboratory mice (Chew et al., 2016; Nelson et al., 2019). Host anti-vector and transgene responses are discussed elsewhere (Sun et al., 2003; Rabinowitz et al., 2019; Wang et al., 2019). Additionally, preexisting adaptive immunity against *S. pyogenes* and *Staphylococcus aureus* Cas9 have also been reported in humans (Charlesworth et al., 2019). However, these findings are unsurprising given the high frequency at which these bacteria infect humans (Lowy, 1998; Roberts et al., 2012). While SpCas9 and SaCas9 remain two of the most broadly used CRISPR enzymes, new orthologues derived from non-pathogenic bacterial species may be required for human therapies where preexisting immunity is a concern. Alternatives such as orthologue specific-epitope engineering or short term suppression (Chew et al., 2016) may theoretically ameliorate immune responses in the short-term. However, the long-term expression of AAV-mediated therapies and their potential for genome insertion at DSB sites (Miller et al., 2004; Hanlon et al., 2019), may limit the feasibility of immunosuppressive approaches.

The low efficiency of precise editing (corrective editing via HDR, prime editing, etc.), in neurons is another significant hurdle for the use of CRISPR for neuroscience research and human therapy. The available data indicate that precise editing occurs at relatively low levels in neurons, limiting the utility of these methods and currently making them unlikely to have any benefit clinically. Although precise editing occurs relatively infrequently in most cell types compared to NHEJ-mediated indel formation, disorders that afflict mitotically active cell populations may be more amenable to HDR-based therapies. For example, hemopoietic progenitor cells can be genetically modified *ex vivo*, clonally selected efficiency, expanded and then re-implanted, essentially bypassing the issue of inefficient HDR mediated precision editing. In another example it was recently demonstrated that AAV-CRISPR could be delivered via tail injection in a mouse model of hereditary tyrosinemia Type 1 (HTI) to correct genetic mutations within the liver (Yin et al., 2014). Although only a small percentage of hepatocytes (<1%) harbored the therapeutic edit, the gene correction provided a fitness advantage to edited cells, which allowed them to repopulate the liver. Unfortunately, given that neurons are postmitotic, precise, CRISPR-mediated editing has limited utility for the foreseeable future until methods are developed to increase its efficiency.

## Conclusions and Future Directions

The CRISPR-Cas system has emerged as a highly adaptable platform with extensive utility in multiple areas of biomedical and basic science. Given its ability to target nearly any gene or RNA transcript, alter gene expression and modify epigenetic states with high specificity, CRISPR-Cas represents an invaluable tool helping drive the rapid pace of discovery in biological sciences. While early studies only demonstrated its use in peripheral tissues, recent efforts have produced CRISPR-Cas systems amenable for use in the CNS. Additionally, the development of CRISPR-expressing animals, as well as the discovery of AAV-compatible orthologues, have provided substantial tools for probing neuronal function at multiple levels of analysis. While newly developed CRISPR-transgenics may be crossed with existing Cre-driving lines, novel CNS-optimized tools will likely require viral vector encoding and delivery. Challenges associated with viral vectors such as packaging constraints, low virus infectivity and low gene editing efficiencies remain limiting factors for using CRISPR in the brain. In order to maximize the therapeutic and research potential of available systems, existing delivery methods must be optimized and new, more effective ways of introducing these systems must be developed. Undoubtedly, future improvements and applications of CRISPR-Cas technology will surface. Despite these challenges, recent advances in CRISPR-Cas technology have provided researchers with powerful new tools for engineering the neuronal genome.

## Synthesis

Reviewing Editor: Christophe Bernard, INSERM & Institut de Neurosciences des Systèmes Decisions are customarily a result of the Reviewing Editor and the peer reviewers coming together and discussing their recommendations until a consensus is reached. When revisions are invited, a fact-based synthesis statement explaining their decision and outlining what is needed to prepare a revision will be listed below. The following reviewer(s) agreed to reveal their identity: Jeremy Day, Ophir Shalem.

Reviewer 1:

This review highlights recent advances in the use of CRISPR-based approaches in the nervous system. In general it is well written and identifies a number of challenges and opportunities in this emerging area. In addition to covering the use of Cas9 for gene editing in various contexts (e.g., to make a transgenic animal, in dividing cell lines, and in mature differentiated neurons), this review also covers approaches based on catalytically inactive dCas9 strategies for gene regulation or epigenetic editing. The review provides a relatively comprehensive overview of current developments in each area, and the illustrations are top notch and enhance the manuscript. Although several other recent reviews and book chapters exist on the same topic (e.g., PMIDs 31191241, 28018138, 31314232, 29259522, 26656253), most of these are now 2-3 years old. Given the rapidly moving nature of this field, this is a timely and useful review. Nevertheless, I have listed several comments for improvement of the manuscript below.

Major suggestions:

1. This review has a chance to extend beyond ground covered by previous reviews by including coverage of more recently described advances in use of CRISPR to understand the nervous system - for example, targeting Cas9 to screen many different genes implicated in neuropsychiatric or neurodegenerative disease (e.g., PMID 30929901), to trace lineage and development of the nervous system (e.g., PMID 29608178), or to isolate functions of individual genes in specific cell types via integration with scRNA-seq (e.g., DOI: https://doi.org/10.1101/791525). These approaches are likely to capture the interests of readers less familiar with these types of approaches, as opposed to a more straightforward (but less novel) repetition of some of the basic features of the CRISPR system.

2. dCas9 sections cover several mouse lines used for CRISPRa and CRISPRi, but are missing some key citations describing these techniques in other contexts (PMIDs 30699347, 27341390, 30863790, 25849986, and 28111074).

3. Figures 3-7 cover similar conceptual ground in use of dCas9 fusion proteins to modify gene expression or local epigenetic states. I would encourage the authors to combine these into a single figure, which would help to show that these approaches are all variations on the same theme, while also preventing some mental redundancy across figures.

4. Figures 3-4 appear to have been skipped in order - Figure 5 is actually the third figure in the manuscript. Additionally, figures should be referenced in the text at appropriate places to alert the reader to graphical illustrations.

5. Page 22, final paragraph: In discussion of the usefulness of multiplexing several different sgRNAs, it would be important to discuss results from Savell, et al (2019 eNeuro; PMID 30863790) since this paper compared effects of multiple sgRNAs at the same locus and also mixed sgRNAs targeted to different genes.

Minor comments:

1. Page 4, paragraph 2: Typo - “predominate” should be “predominant”

2. Page 9, second paragraph: Insert period after “... when conjugated to other proteins”

3. Page 12, last paragraph: Platt, et al citation repeated twice.

4. Page 15, second paragraph: de Solis, et al., citation should not be in parentheses

5. Page 25, second line: “predominate” should be “predominant”

Reviewer 2:

This topic of this review, genome-editing in neurons, is important, timely, and a good review that focuses specifically on the challenges of applying CRISPR based perturbations in neuronal cells will be very useful, because while the development and applications of CRISPR associated proteins is covered in many excellent reviews, very few of those focus specifically on editing in neuronal cells. Unfortunately, the current review reads more like “Genome editing in neuroscience research”. The sections that discuss genome-editing in the CNS are good. But the review as a whole is quite unfocused, the sections that discuss the general development of CRISPR systems for different applications are highly redundant with other published reviews and the sections that discuss editing in the CNS lacks a few important points of discussion. I suggest a major revision to reorganize the paper and focus much more on the major challenges of neuronal editing for both therapeutic and research applications.

Specific comments:

• About 40-50% of the paper (I did not precisely measure this) discusses general development of CRISPR genetic perturbation systems. This can probably be shortened with references to existing published reviews.

• This review only considers precise editing in neuronal cells mediated by HDR. Other approaches exist especially for research applications. NHEJ mediated editing in non-dividing cells has been demonstrated for gene corrections (PMID: 27851729) and gene tagging (PMID: 31272828). The enzyme Cpf1 is also worth mentioning in this regard as its staggered cutting might enable gene insertions that are independent of HDR. Lastly, methods that do not relay on the generation of a DSB as based editors and the most recent prime editors should be discussed.

• A discussion of the differences in DNA repair pathways in dividing vs. neuronal cells would be useful here.

• Choice of titles for the different sections is a bit confusing, for example after "Genome Editing in the mammalian brain" (which is a similar title to the article title) discusses viral delivery into the brain. This section is preceded by a discussion on RNP delivery. This should all be under a much more extensive discussion on the delivery challenges into the CNS with a separation between clinical and research applications.

• There is an extensive discussion on germline editing for the generation of transgenic animals. If the theme of the article is neuronal editing this seems irrelevant. If this is indeed a review on genome-editing in neuroscience then this section is useful, especially the discussion of rat models which are less discussed in other reviews.

• In the discussion of inducible systems, it is probably worth mentioning this paper: PMID: 25848930 that resulted in a much better sensitivity compared to Tet based systems.

• Especially as this is a review article, the authors should make sure that (1) all statements are backed by citations. (2) All citations are correct. Example of (1): In page 13 the paragraph starting with "While CRISPR-Cas9 has been used to edit a bevy of cell types and tissues..." contains no citations at all. Example of (2): "and gain-of-function (GOF) screens including a recent report of a successful genome-wide screen in the brain (Chow et al., 2017; Cong et al., 2013). - Wrong reference" Cong et al. 2013 does not describe a genome-wide screen.

• PMID: 31422865 should be discussed.

• A summary of the main challenges: delivery, off target activity, immunogenicity of CRISPR proteins, efficiency of precise editing in neurons would be useful to add in the last section.

## References

[B1] Abudayyeh OO, Gootenberg JS, Konermann S, Joung J, Slaymaker IM, Cox DB, Shmakov S, Makarova KS, Semenova E, Minakhin L, Severinov K, Regev A, Lander ES, Koonin EV, Zhang F (2016) C2c2 is a single-component programmable RNA-guided RNA-targeting CRISPR effector. Science 353:aaf5573. 10.1126/science.aaf5573 27256883 PMC5127784

[B2] Abudayyeh OO, Gootenberg JS, Essletzbichler P, Han S, Joung J, Belanto JJ, Verdine V, Cox DBT, Kellner MJ, Regev A, Lander ES, Voytas DF, Ting AY, Zhang F (2017) RNA targeting with CRISPR-Cas13. Nature 550:280–284. 10.1038/nature24049 28976959 PMC5706658

[B3] Anzalone AV, Randolph PB, Davis JR, Sousa AA, Koblan LW, Levy JM, Chen PJ, Wilson C, Newby GA, Raguram A, Liu DR (2019) Search-and-replace genome editing without double-strand breaks or donor DNA. Nature 576:149–157. 10.1038/s41586-019-1711-4 31634902 PMC6907074

[B4] Bäck S, Necarsulmer J, Whitaker LR, Coke LM, Koivula P, Heathward EJ, Fortuno LV, Zhang Y, Yeh CG, Baldwin HA, Spencer MD, Mejias-Aponte CA, Pickel J, Hoffman AF, Spivak CE, Lupica CR, Underhill SM, Amara SG, Domanskyi A, Anttila JE, et al (2019) Neuron-specific genome modification in the adult rat brain using CRISPR-Cas9 transgenic rats. Neuron 102:105–119.e8. 30792150 10.1016/j.neuron.2019.01.035 PMC11805586

[B5] Behm M, Öhman M (2016) RNA editing: a contributor to neuronal dynamics in the mammalian brain. Trends Genet 32:165–175. 10.1016/j.tig.2015.12.005 26803450

[B6] Boeve BF, Hutton M (2008) Refining frontotemporal dementia with parkinsonism linked to chromosome 17: introducing FTDP-17 (MAPT) and FTDP-17 (PGRN). Arch Neurol 65:460–464. 10.1001/archneur.65.4.460 18413467 PMC2746630

[B7] Butler MG (2009) Genomic imprinting disorders in humans: a mini-review. J Assist Reprod Genet 26:477–486. 10.1007/s10815-009-9353-3 19844787 PMC2788689

[B8] Cameron P, Coons MM, Klompe SE, Lied AM, Smith SC, Vidal B, Donohoue PD, Rotstein T, Kohrs BW, Nyer DB, Kennedy R, Banh LM, Williams C, Toh MS, Irby MJ, Edwards LS, Lin CH, Owen ALG, Künne T, van der Oost J, et al. (2019) Harnessing type I CRISPR-Cas systems for genome engineering in human cells. Nat Biotechnol 37:1471–1477. 10.1038/s41587-019-0310-0 31740839

[B9] Campa CC, Weisbach NR, Santinha AJ, Incarnato D, Platt RJ (2019) Multiplexed genome engineering by Cas12a and CRISPR arrays encoded on single transcripts. Nat Methods 16:887–893. 10.1038/s41592-019-0508-6 31406383

[B10] Capecchi MR (2005) Gene targeting in mice: functional analysis of the mammalian genome for the twenty-first century. Nat Rev Genet 6:507–512. 10.1038/nrg1619 15931173

[B11] Carroll D (2011) Genome engineering with zinc-finger nucleases. Genetics 188:773–782. 10.1534/genetics.111.131433 21828278 PMC3176093

[B12] Casini A, Olivieri M, Petris G, Montagna C, Reginato G, Maule G, Lorenzin F, Prandi D, Romanel A, Demichelis F, Inga A, Cereseto A (2018) A highly specific SpCas9 variant is identified by in vivo screening in yeast. Nat Biotechnol 36:265–271. 10.1038/nbt.4066 29431739 PMC6066108

[B13] Castanotto D, Rossi JJ (2009) The promises and pitfalls of RNA-interference-based therapeutics. Nature 457:426–433. 10.1038/nature07758 19158789 PMC2702667

[B14] Chan KY, Jang MJ, Yoo BB, Greenbaum A, Ravi N, Wu WL, Sánchez-Guardado L, Lois C, Mazmanian SK, Deverman BE, Gradinaru V (2017) Engineered AAVs for efficient noninvasive gene delivery to the central and peripheral nervous systems. Nat Neurosci 20:1172–1179. 10.1038/nn.4593 28671695 PMC5529245

[B15] Chapman KM, Medrano GA, Jaichander P, Chaudhary J, Waits AE, Nobrega MA, Hotaling JM, Ober C, Hamra FK (2015) Targeted germline modifications in rats using CRISPR/Cas9 and spermatogonial stem cells. Cell Rep 10:1828–1835. 10.1016/j.celrep.2015.02.040 25772367 PMC4376630

[B16] Charlesworth CT, Deshpande PS, Dever DP, Camarena J, Lemgart VT, Cromer MK, Vakulskas CA, Collingwood MA, Zhang L, Bode NM, Behlke MA, Dejene B, Cieniewicz B, Romano R, Lesch BJ, Gomez-Ospina N, Mantri S, Pavel-Dinu M, Weinberg KI, Porteus MH (2019) Identification of preexisting adaptive immunity to Cas9 proteins in humans. Nat Med 25:249–254. 10.1038/s41591-018-0326-x 30692695 PMC7199589

[B17] Chatterjee P, Jakimo N, Jacobson JM (2018) Minimal PAM specificity of a highly similar SpCas9 ortholog. Sci Adv 4:eaau0766. 10.1126/sciadv.aau0766 30397647 PMC6200363

[B18] Chavez A, Scheiman J, Vora S, Pruitt BW, Tuttle M, P R Iyer E, Lin S, Kiani S, Guzman CD, Wiegand DJ, Ter-Ovanesyan D, Braff JL, Davidsohn N, Housden BE, Perrimon N, Weiss R, Aach J, Collins JJ, Church GM (2015) Highly efficient Cas9-mediated transcriptional programming. Nat Methods 12:326–328. 10.1038/nmeth.3312 25730490 PMC4393883

[B19] Chavez A, Tuttle M, Pruitt BW, Ewen-Campen B, Chari R, Ter-Ovanesyan D, Haque SJ, Cecchi RJ, Kowal EJK, Buchthal J, Housden BE, Perrimon N, Collins JJ, Church G (2016) Comparison of Cas9 activators in multiple species. Nat Methods 13:563–567. 10.1038/nmeth.3871 27214048 PMC4927356

[B20] Chen JS, Dagdas YS, Kleinstiver BP, Welch MM, Sousa AA, Harrington LB, Sternberg SH, Joung JK, Yildiz A, Doudna JA (2017) Enhanced proofreading governs CRISPR-Cas9 targeting accuracy. Nature 550:407–410. 10.1038/nature24268 28931002 PMC5918688

[B21] Chen LF, Lin YT, Gallegos DA, Hazlett MF, Gómez-Schiavon M, Yang MG, Kalmeta B, Zhou AS, Holtzman L, Gersbach CA, Grandl J, Buchler NE, West AE (2019) Enhancer histone acetylation modulates transcriptional bursting dynamics of neuronal activity-inducible genes. Cell Rep 26:1174–1188.e5. 10.1016/j.celrep.2019.01.032 30699347 PMC6376993

[B22] Chew WL, Tabebordbar M, Cheng JK, Mali P, Wu EY, Ng AH, Zhu K, Wagers AJ, Church GM (2016) A multifunctional AAV-CRISPR-Cas9 and its host response. Nat Methods 13:868–874. 10.1038/nmeth.3993 27595405 PMC5374744

[B23] Chong ZS, Ohnishi S, Yusa K, Wright GJ (2018) Pooled extracellular receptor-ligand interaction screening using CRISPR activation. Genome Biol 19:205. 10.1186/s13059-018-1581-3 30477585 PMC6258485

[B24] Chow RD, Guzman CD, Wang G, Schmidt F, Youngblood MW, Ye L, Errami Y, Dong MB, Martinez MA, Zhang S, Renauer P, Bilguvar K, Gunel M, Sharp PA, Zhang F, Platt RJ, Chen S (2017) AAV-mediated direct in vivo CRISPR screen identifies functional suppressors in glioblastoma. Nat Neurosci 20:1329–1341. 10.1038/nn.4620 28805815 PMC5614841

[B25] Chu VT, Weber T, Wefers B, Wurst W, Sander S, Rajewsky K, Kühn R (2015) Increasing the efficiency of homology-directed repair for CRISPR-Cas9-induced precise gene editing in mammalian cells. Nat Biotechnol 33:543–548. 10.1038/nbt.3198 25803306

[B26] Chung CY, Seo H, Sonntag KC, Brooks A, Lin L, Isacson O (2005) Cell type-specific gene expression of midbrain dopaminergic neurons reveals molecules involved in their vulnerability and protection. Hum Mol Genet 14:1709–1725. 10.1093/hmg/ddi178 15888489 PMC2674782

[B27] Colella P, Ronzitti G, Mingozzi F (2018) Emerging issues in AAV-mediated in vivo gene therapy. Mol Ther Methods Clin Dev 8:87–104. 10.1016/j.omtm.2017.11.007 29326962 PMC5758940

[B28] Cong L, Ran FA, Cox D, Lin S, Barretto R, Habib N, Hsu PD, Wu X, Jiang W, Marraffini LA, Zhang F (2013) Multiplex genome engineering using CRISPR/Cas systems. Science 339:819–823. 10.1126/science.1231143 23287718 PMC3795411

[B29] Cox DB, Platt RJ, Zhang F (2015) Therapeutic genome editing: prospects and challenges. Nat Med 21:121–131. 10.1038/nm.3793 25654603 PMC4492683

[B30] Cox DBT, Gootenberg JS, Abudayyeh OO, Franklin B, Kellner MJ, Joung J, Zhang F (2017) RNA editing with CRISPR-Cas13. Science 358:1019–1027. 10.1126/science.aaq0180 29070703 PMC5793859

[B31] Davis KM, Pattanayak V, Thompson DB, Zuris JA, Liu DR (2015) Small molecule-triggered Cas9 protein with improved genome-editing specificity. Nat Chem Biol 11:316–318. 10.1038/nchembio.1793 25848930 PMC4402137

[B32] de Solis CA, Holehonnur R, Banerjee A, Luong JA, Lella SK, Ho A, Pahlavan B, Ploski JE (2015) Viral delivery of shRNA to amygdala neurons leads to neurotoxicity and deficits in Pavlovian fear conditioning. Neurobiol Learn Mem 124:34–47. 10.1016/j.nlm.2015.07.005 26182988 PMC4568141

[B33] de Solis CA, Ho A, Holehonnur R, Ploski JE (2016) The development of a viral mediated CRISPR/Cas9 system with doxycycline dependent gRNA expression for inducible in vitro and in vivo genome editing. Front Mol Neurosci 9:70. 10.3389/fnmol.2016.00070 27587996 PMC4988984

[B34] Dixit A, Parnas O, Li B, Chen J, Fulco CP, Jerby-Arnon L, Marjanovic ND, Dionne D, Burks T, Raychowdhury R, Adamson B, Norman TM, Lander ES6, Weissman JS, Friedman N, Regev A (2016) Perturb-Seq: Dissecting Molecular Circuits with Scalable Single-Cell RNA Profiling of Pooled Genetic Screens. Cell 167:1853–1866.e17. 27984732 10.1016/j.cell.2016.11.038 PMC5181115

[B35] Doench JG, Fusi N, Sullender M, Hegde M, Vaimberg EW, Donovan KF, Smith I, Tothova Z, Wilen C, Orchard R, Virgin HW, Listgarten J, Root DE (2016) Optimized sgRNA design to maximize activity and minimize off-target effects of CRISPR-Cas9. Nat Biotechnol 34:184–191. 10.1038/nbt.3437 26780180 PMC4744125

[B36] Dominguez AA, Lim WA, Qi LS (2016) Beyond editing: repurposing CRISPR-Cas9 for precision genome regulation and interrogation. Nat Rev Mol Cell Biol 17:5–15. 10.1038/nrm.2015.2 26670017 PMC4922510

[B37] Dow LE, Fisher J, O'Rourke KP, Muley A, Kastenhuber ER, Livshits G, Tschaharganeh DF, Socci ND, Lowe SW (2015) Inducible in vivo genome editing with CRISPR-Cas9. Nat Biotechnol 33:390–394. 10.1038/nbt.3155 25690852 PMC4390466

[B38] Ellenbroek B, Youn J (2016) Rodent models in neuroscience research: is it a rat race? Dis Model Mech 9:1079–1087. 10.1242/dmm.026120 27736744 PMC5087838

[B39] Frank CL, Liu F, Wijayatunge R, Song L, Biegler MT, Yang MG, Vockley CM, Safi A, Gersbach CA, Crawford GE, West AE (2015) Regulation of chromatin accessibility and Zic binding at enhancers in the developing cerebellum. Nat Neurosci 18:647–656. 10.1038/nn.3995 25849986 PMC4414887

[B40] Gaj T, Gersbach CA, Barbas CF 3rd (2013) ZFN, TALEN, and CRISPR/Cas-based methods for genome engineering. Trends Biotechnol 31:397–405. 10.1016/j.tibtech.2013.04.004 23664777 PMC3694601

[B41] Gilbert LA, Larson MH, Morsut L, Liu Z, Brar GA, Torres SE, Stern-Ginossar N, Brandman O, Whitehead EH, Doudna JA, Lim WA, Weissman JS, Qi LS (2013) CRISPR-mediated modular RNA-guided regulation of transcription in eukaryotes. Cell 154:442–451. 10.1016/j.cell.2013.06.044 23849981 PMC3770145

[B42] Gossen M, Bujard H (1992) Tight control of gene expression in mammalian cells by tetracycline-responsive promoters. Proc Natl Acad Sci USA 89:5547–5551. 10.1073/pnas.89.12.5547 1319065 PMC49329

[B43] Gossen M, Freundlieb S, Bender G, Müller G, Hillen W, Bujard H (1995) Transcriptional activation by tetracyclines in mammalian cells. Science 268:1766–1769. 10.1126/science.7792603 7792603

[B44] Gray JA, Shi Y, Usui H, During MJ, Sakimura K, Nicoll RA (2011) Distinct modes of AMPA receptor suppression at developing synapses by GluN2A and GluN2B: single-cell NMDA receptor subunit deletion in vivo. Neuron 71:1085–1101. 10.1016/j.neuron.2011.08.007 21943605 PMC3183990

[B45] Guilbaud M, Devaux M, Couzinié C, Le Duff J, Toromanoff A, Vandamme C, Jaulin N, Gernoux G, Larcher T, Moullier P, Le Guiner C, Adjali O (2019) Five years of successful inducible transgene expression following locoregional adeno-associated virus delivery in nonhuman primates with no detectable immunity. Hum Gene Ther 30:802–813. 10.1089/hum.2018.234 30808235 PMC6648187

[B46] Haggerty DL, Grecco GG, Reeves KC, Atwood B (2020) Adeno-associated viral vectors in neuroscience research. Mol Ther Methods Clin Dev 17:69–82. 10.1016/j.omtm.2019.11.012 31890742 PMC6931098

[B47] Hanlon KS, Kleinstiver BP, Garcia SP, Zaborowski MP, Volak A, Spirig SE, Muller A, Sousa AA, Tsai SQ, Bengtsson NE, Lööv C, Ingelsson M, Chamberlain JS, Corey DP, Aryee MJ, Joung JK, Breakefield XO, Maguire CA, György B (2019) High levels of AAV vector integration into CRISPR-induced DNA breaks. Nat Commun 10:4439. 10.1038/s41467-019-12449-2 31570731 PMC6769011

[B48] Hart T, Chandrashekhar M, Aregger M, Steinhart Z, Brown KR, MacLeod G, Mis M, Zimmermann M, Fradet-Turcotte A, Sun S, Mero P, Dirks P, Sidhu S, Roth FP, Rissland OS, Durocher D, Angers S, Moffat J (2015) High-resolution CRISPR screens reveal fitness genes and genotype-specific cancer liabilities. Cell 163:1515–1526. 10.1016/j.cell.2015.11.015 26627737

[B49] Heerboth S, Lapinska K, Snyder N, Leary M, Rollinson S, Sarkar S (2014) Use of epigenetic drugs in disease: an overview. Genet Epigenet 6:9–19. 10.4137/GEG.S12270 25512710 PMC4251063

[B50] Heman-Ackah SM, Bassett AR, Wood MJ (2016) Precision modulation of neurodegenerative disease-related gene expression in human iPSC-derived neurons. Sci Rep 6:28420. 10.1038/srep28420 27341390 PMC4920027

[B51] Henao-Mejia J, Williams A, Rongvaux A, Stein J, Hughes C, Flavell RA (2016) Generation of genetically modified mice using the CRISPR-Cas9 genome-editing system. Cold Spring Harbor Protoc 2016:pdb.prot090704. 10.1101/pdb.prot090704 26832688 PMC4905559

[B52] Hilton IB, D'Ippolito AM, Vockley CM, Thakore PI, Crawford GE, Reddy TE, Gersbach CA (2015) Epigenome editing by a CRISPR-Cas9-based acetyltransferase activates genes from promoters and enhancers. Nat Biotechnol 33:510–517. 10.1038/nbt.3199 25849900 PMC4430400

[B53] Horlbeck MA, Gilbert LA, Villalta JE, Adamson B, Pak RA, Chen Y, Fields AP, Park CY, Corn JE, Kampmann M, Weissman JS (2016) Compact and highly active next-generation libraries for CRISPR-mediated gene repression and activation. Elife 5 10.7554/eLife.19760 PMC5094855 27661255

[B54] Hu JH, Miller SM, Geurts MH, Tang W, Chen L, Sun N, Zeina CM, Gao X, Rees HA, Lin Z, Liu DR (2018) Evolved Cas9 variants with broad PAM compatibility and high DNA specificity. Nature 556:57–63. 10.1038/nature26155 29512652 PMC5951633

[B55] Huang YA, Zhou B, Wernig M, Südhof TC (2017) ApoE2, ApoE3, and ApoE4 differentially stimulate APP transcription and Aβ secretion. Cell 168:427–441.e21. 10.1016/j.cell.2016.12.044 28111074 PMC5310835

[B56] Incontro S, Asensio CS, Edwards RH, Nicoll RA (2014) Efficient, complete deletion of synaptic proteins using CRISPR. Neuron 83:1051–1057. 10.1016/j.neuron.2014.07.043 25155957 PMC4195490

[B57] Jackson AL, Linsley PS (2010) Recognizing and avoiding siRNA off-target effects for target identification and therapeutic application. Nat Rev Drug Discov 9:57–67. 10.1038/nrd3010 20043028

[B58] Jin X, Simmons SK, Guo AX, Shetty AS, Ko M, Nguyen L, Robinson E, Oyler P, Curry N, Deangeli G, Lodato S, Levin JZ, Regev A, Zhang F, Arlotta P (2019) In vivo Perturb-seq reveals neuronal and glial abnormalities associated with autism risk genes. bioRxiv 791525. 10.1126/science.aaz6063 PMC7985844 33243861

[B59] Jinek M, Chylinski K, Fonfara I, Hauer M, Doudna JA, Charpentier E (2012) A programmable dual-RNA-guided DNA endonuclease in adaptive bacterial immunity. Science 337:816–821. 10.1126/science.1225829 22745249 PMC6286148

[B60] Joung J, Konermann S, Gootenberg JS, Abudayyeh OO, Platt RJ, Brigham MD, Sanjana NE, Zhang F (2017) Genome-scale CRISPR-Cas9 knockout and transcriptional activation screening. Nat Protoc 12:828–863. 10.1038/nprot.2017.016 28333914 PMC5526071

[B61] Joung JK, Sander JD (2013) TALENs: a widely applicable technology for targeted genome editing. Nat Rev Mol Cell Biol 14:49–55. 10.1038/nrm3486 23169466 PMC3547402

[B62] Kawano F, Suzuki H, Furuya A, Sato M (2015) Engineered pairs of distinct photoswitches for optogenetic control of cellular proteins. Nat Commun 6:6256. 10.1038/ncomms7256 25708714

[B63] Kim YG, Cha J, Chandrasegaran S (1996) Hybrid restriction enzymes: zinc finger fusions to Fok I cleavage domain. Proc Natl Acad Sci USA 93:1156–1160. 10.1073/pnas.93.3.1156 8577732 PMC40048

[B64] Kleinstiver BP, Prew MS, Tsai SQ, Topkar VV, Nguyen NT, Zheng Z, Gonzales AP, Li Z, Peterson RT, Yeh JR, Aryee MJ, Joung JK (2015) Engineered CRISPR-Cas9 nucleases with altered PAM specificities. Nature 523:481–485. 10.1038/nature14592 26098369 PMC4540238

[B65] Kleinstiver BP, Pattanayak V, Prew MS, Tsai SQ, Nguyen NT, Zheng Z, Joung JK (2016) High-fidelity CRISPR-Cas9 nucleases with no detectable genome-wide off-target effects. Nature 529:490–495. 10.1038/nature16526 26735016 PMC4851738

[B66] Konermann S, Brigham MD, Trevino AE, Joung J, Abudayyeh OO, Barcena C, Hsu PD, Habib N, Gootenberg JS, Nishimasu H, Nureki O, Zhang F (2015) Genome-scale transcriptional activation by an engineered CRISPR-Cas9 complex. Nature 517:583–588. 10.1038/nature14136 25494202 PMC4420636

[B67] Konermann S, Lotfy P, Brideau NJ, Oki J, Shokhirev MN, Hsu PD (2018) Transcriptome engineering with RNA-targeting type VI-D CRISPR effectors. Cell 173:665–676.e4. 10.1016/j.cell.2018.02.033 29551272 PMC5910255

[B68] Kumar N, Stanford W, de Solis C, Aradhana, Abraham ND, Dao TJ, Thaseen S, Sairavi A, Gonzalez CU, Ploski JE (2018) The development of an AAV-based CRISPR SaCas9 genome editing system that can be delivered to neurons in vivo and regulated via doxycycline and Cre-recombinase. Front Mol Neurosci 11:413. 10.3389/fnmol.2018.00413 30483052 PMC6243075

[B69] Kuscu C, Arslan S, Singh R, Thorpe J, Adli M (2014) Genome-wide analysis reveals characteristics of off-target sites bound by the Cas9 endonuclease. Nat Biotechnol 32:677–683. 10.1038/nbt.2916 24837660

[B70] LaBar KS, Cabeza R (2006) Cognitive neuroscience of emotional memory. Nat Rev Neurosci 7:54–64. 10.1038/nrn1825 16371950

[B71] Lee B, Lee K, Panda S, Gonzales-Rojas R, Chong A, Bugay V, Park HM, Brenner R, Murthy N, Lee HY (2018) Nanoparticle delivery of CRISPR into the brain rescues a mouse model of fragile X syndrome from exaggerated repetitive behaviours. Nat Biomed Eng 2:497–507. 10.1038/s41551-018-0252-8 30948824 PMC6544395

[B72] Lei Y, Zhang X, Su J, Jeong M, Gundry MC, Huang YH, Zhou Y, Li W, Goodell MA (2017) Targeted DNA methylation in vivo using an engineered dCas9-MQ1 fusion protein. Nat Commun 8:16026. 10.1038/ncomms16026 28695892 PMC5508226

[B73] Lentz TB, Gray SJ, Samulski RJ (2012) Viral vectors for gene delivery to the central nervous system. Neurobiol Dis 48:179–188. 10.1016/j.nbd.2011.09.014 22001604 PMC3293995

[B74] Li W, Teng F, Li T, Zhou Q (2013) Simultaneous generation and germline transmission of multiple gene mutations in rat using CRISPR-Cas systems. Nat Biotechnol 31:684–686. 10.1038/nbt.2652 23929337

[B75] Liu J, Gaj T, Wallen MC, Barbas CF 3rd (2015) Improved cell-penetrating zinc-finger nuclease proteins for precision genome engineering. Mol Ther Nucleic Acids 4:e232. 10.1038/mtna.2015.6 25756962 PMC4354341

[B76] Liu J, Srinivasan S, Li CY, Ho IL, Rose J, Shaheen M, Wang G, Yao W, Deem A, Bristow C, Hart T, Draetta G (2019) Pooled library screening with multiplexed Cpf1 library. Nat Commun 10:3144. 10.1038/s41467-019-10963-x 31316073 PMC6637147

[B77] Liu KI, Ramli MN, Woo CW, Wang Y, Zhao T, Zhang X, Yim GR, Chong BY, Gowher A, Chua MZ, Jung J, Lee JH, Tan MH (2016a) A chemical-inducible CRISPR-Cas9 system for rapid control of genome editing. Nat Chem Biol 12:980–987. 10.1038/nchembio.2179 27618190

[B78] Liu XS, Wu H, Ji X, Stelzer Y, Wu X, Czauderna S, Shu J, Dadon D, Young RA, Jaenisch R (2016b) Editing DNA methylation in the mammalian genome. Cell 167:233–247.e7. 10.1016/j.cell.2016.08.056 27662091 PMC5062609

[B79] Liu SJ, Horlbeck MA, Cho SW, Birk HS, Malatesta M, He D, Attenello FJ, Villalta JE, Cho MY, Chen Y, Mandegar MA, Olvera MP, Gilbert LA, Conklin BR, Chang HY, Weissman JS, Lim DA (2017) CRISPRi-based genome-scale identification of functional long noncoding RNA loci in human cells. Science 355:eaah7111 10.1126/science.aah7111 PMC5394926 27980086

[B80] Liu XS, Wu H, Krzisch M, Wu X, Graef J, Muffat J, Hnisz D, Li CH, Yuan B, Xu C, Li Y, Vershkov D, Cacace A, Young RA, Jaenisch R (2018a) Rescue of fragile X syndrome neurons by DNA methylation editing of the FMR1 gene. Cell 172:979–992.e6. 10.1016/j.cell.2018.01.012 29456084 PMC6375087

[B81] Liu Y, Yu C, Daley TP, Wang F, Cao WS, Bhate S, Lin X, Still C 2nd , Liu H, Zhao D, Wang H, Xie XS, Ding S, Wong WH, Wernig M, Qi LS (2018b) CRISPR activation screens systematically identify factors that drive neuronal fate and reprogramming. Cell Stem Cell 23:758–771.e8. 10.1016/j.stem.2018.09.003 30318302 PMC6214761

[B82] Long C, Amoasii L, Mireault AA, McAnally JR, Li H, Sanchez-Ortiz E, Bhattacharyya S, Shelton JM, Bassel-Duby R, Olson EN (2016) Postnatal genome editing partially restores dystrophin expression in a mouse model of muscular dystrophy. Science 351:400–403. 10.1126/science.aad5725 26721683 PMC4760628

[B83] Lowy FD (1998) *Staphylococcus aureus* infections. N Engl J Med 339:520–532. 10.1056/NEJM199808203390806 9709046

[B84] Maeder ML, Linder SJ, Cascio VM, Fu Y, Ho QH, Joung JK (2013) CRISPR RNA-guided activation of endogenous human genes. Nat Methods 10:977–979. 10.1038/nmeth.2598 23892898 PMC3794058

[B85] Mali P, Yang L, Esvelt KM, Aach J, Guell M, DiCarlo JE, Norville JE, Church GM (2013) RNA-guided human genome engineering via Cas9. Science 339:823–826. 10.1126/science.1232033 23287722 PMC3712628

[B86] Maruyama T, Dougan SK, Truttmann MC, Bilate AM, Ingram JR, Ploegh HL (2015) Increasing the efficiency of precise genome editing with CRISPR-Cas9 by inhibition of nonhomologous end joining. Nat Biotechnol 33:538–542. 10.1038/nbt.3190 25798939 PMC4618510

[B87] Matera AG, Wang Z (2014) A day in the life of the spliceosome. Nat Rev Mol Cell Biol 15:108–121. 10.1038/nrm3742 24452469 PMC4060434

[B88] McKenna A, Findlay GM, Gagnon JA, Horwitz MS, Schier AF, Shendure J (2016) Whole-organism lineage tracing by combinatorial and cumulative genome editing. Science 353:aaf7907. 10.1126/science.aaf7907 27229144 PMC4967023

[B89] Mikuni T, Nishiyama J, Sun Y, Kamasawa N, Yasuda R (2016) High-throughput, high-resolution mapping of protein localization in mammalian brain by in vivo genome editing. Cell 165:1803–1817. 10.1016/j.cell.2016.04.044 27180908 PMC4912470

[B90] Miller DG, Petek LM, Russell DW (2004) Adeno-associated virus vectors integrate at chromosome breakage sites. Nat Genet 36:767–773. 10.1038/ng1380 15208627

[B91] Morgens DW, Wainberg M, Boyle EA, Ursu O, Araya CL, Tsui CK, Haney MS, Hess GT, Han K, Jeng EE, Li A, Snyder MP, Greenleaf WJ, Kundaje A, Bassik MC (2017) Genome-scale measurement of off-target activity using Cas9 toxicity in high-throughput screens. Nat Commun 8:15178. 10.1038/ncomms15178 28474669 PMC5424143

[B92] Nathwani AC, Tuddenham EGD, Rangarajan S, Rosales C, McIntosh J, Linch DC, Chowdary P, Riddell A, Pie AJ, Harrington C, O'Beirne J, Smith K, Pasi J, Glader B, Rustagi P, Ng CYC, Kay MA, Zhou J, Spence Y, Morton CL, et al. (2011) Adenovirus-associated virus vector-mediated gene transfer in hemophilia B. N Engl J Med 365:2357–2365. 10.1056/NEJMoa1108046 22149959 PMC3265081

[B93] Nelson CE, Wu Y, Gemberling MP, Oliver ML, Waller MA, Bohning JD, Robinson-Hamm JN, Bulaklak K, Castellanos Rivera RM, Collier JH, Asokan A, Gersbach CA (2019) Long-term evaluation of AAV-CRISPR genome editing for Duchenne muscular dystrophy. Nat Med 25:427–432. 10.1038/s41591-019-0344-3 30778238 PMC6455975

[B94] Nihongaki Y, Kawano F, Nakajima T, Sato M (2015) Photoactivatable CRISPR-Cas9 for optogenetic genome editing. Nat Biotechnol 33:755–760. 10.1038/nbt.3245 26076431

[B95] Nishiyama J, Mikuni T, Yasuda R (2017) Virus-mediated genome editing via homology-directed repair in mitotic and postmitotic cells in mammalian brain. Neuron 96:755–768.e5. 10.1016/j.neuron.2017.10.004 29056297 PMC5691606

[B96] Ousterout DG, Kabadi AM, Thakore PI, Majoros WH, Reddy TE, Gersbach CA (2015) Multiplex CRISPR/Cas9-based genome editing for correction of dystrophin mutations that cause Duchenne muscular dystrophy. Nat Commun 6:6244. 10.1038/ncomms7244 25692716 PMC4335351

[B97] Park H, Oh J, Shim G, Cho B, Chang Y, Kim S, Baek S, Kim H, Shin J, Choi H, Yoo J, Kim J, Jun W, Lee M, Lengner CJ, Oh YK, Kim J (2019) In vivo neuronal gene editing via CRISPR-Cas9 amphiphilic nanocomplexes alleviates deficits in mouse models of Alzheimer’s disease. Nat Neurosci 22:524–528. 10.1038/s41593-019-0352-0 30858603

[B98] Persico AM, Napolioni V (2013) Autism genetics. Behav Brain Res 251:95–112. 10.1016/j.bbr.2013.06.012 23769996

[B99] Pickar-Oliver A, Gersbach CA (2019) The next generation of CRISPR-Cas technologies and applications. Nat Rev Mol Cell Biol 20:490–507. 10.1038/s41580-019-0131-5 31147612 PMC7079207

[B100] Pickar-Oliver A, Black JB, Lewis MM, Mutchnick KJ, Klann TS, Gilcrest KA, Sitton MJ, Nelson CE, Barrera A, Bartelt LC, Reddy TE, Beisel CL, Barrangou R, Gersbach CA (2019) Targeted transcriptional modulation with type I CRISPR-Cas systems in human cells. Nat Biotechnol 37:1493–1501. 10.1038/s41587-019-0235-7 31548729 PMC6893126

[B101] Platt RJ, Chen S, Zhou Y, Yim MJ, Swiech L, Kempton HR, Dahlman JE, Parnas O, Eisenhaure TM, Jovanovic M, Graham DB, Jhunjhunwala S, Heidenreich M, Xavier RJ, Langer R, Anderson DG, Hacohen N, Regev A, Feng G, Sharp PA, Zhang F (2014) CRISPR-Cas9 knockin mice for genome editing and cancer modeling. Cell 159:440–455. 10.1016/j.cell.2014.09.014 25263330 PMC4265475

[B102] Polstein LR, Gersbach CA (2015) A light-inducible CRISPR-Cas9 system for control of endogenous gene activation. Nat Chem Biol 11:198–200. 10.1038/nchembio.1753 25664691 PMC4412021

[B103] Qi LS, Larson MH, Gilbert LA, Doudna JA, Weissman JS, Arkin AP, Lim WA (2013) Repurposing CRISPR as an RNA-guided platform for sequence-specific control of gene expression. Cell 152:1173–1183. 10.1016/j.cell.2013.02.022 23452860 PMC3664290

[B104] Rabinowitz J, Chan YK, Samulski RJ (2019) Adeno-associated virus (AAV) versus immune response. Viruses 11:102 10.3390/v11020102 PMC6409805 30691064

[B105] Raj B, Wagner DE, McKenna A, Pandey S, Klein AM, Shendure J, Gagnon JA, Schier AF (2018) Simultaneous single-cell profiling of lineages and cell types in the vertebrate brain. Nat Biotechnol 36:442–450. 10.1038/nbt.4103 29608178 PMC5938111

[B106] Ran FA, Cong L, Yan WX, Scott DA, Gootenberg JS, Kriz AJ, Zetsche B, Shalem O, Wu X, Makarova KS, Koonin EV, Sharp PA, Zhang F (2015) In vivo genome editing using *Staphylococcus aureus* Cas9. Nature 520:186–191. 10.1038/nature14299 25830891 PMC4393360

[B107] Rees HA, Liu DR (2018) Base editing: precision chemistry on the genome and transcriptome of living cells. Nat Rev Genet 19:770–788. 10.1038/s41576-018-0059-1 30323312 PMC6535181

[B108] Remy S, Chenouard V, Tesson L, Usal C, Ménoret S, Brusselle L, Heslan JM, Nguyen TH, Bellien J, Merot J, De Cian A, Giovannangeli C, Concordet JP, Anegon I (2017) Generation of gene-edited rats by delivery of CRISPR/Cas9 protein and donor DNA into intact zygotes using electroporation. Sci Rep 7:16554. 10.1038/s41598-017-16328-y 29185448 PMC5707420

[B109] Roberts AL, Connolly KL, Kirse DJ, Evans AK, Poehling KA, Peters TR, Reid SD (2012) Detection of group A *Streptococcus* in tonsils from pediatric patients reveals high rate of asymptomatic streptococcal carriage. BMC Pediatr 12:3. 10.1186/1471-2431-12-3 22230361 PMC3279307

[B110] Saleh-Gohari N, Helleday T (2004) Conservative homologous recombination preferentially repairs DNA double-strand breaks in the S phase of the cell cycle in human cells. Nucleic Acids Res 32:3683–3688. 10.1093/nar/gkh703 15252152 PMC484186

[B111] Sanjana NE, Shalem O, Zhang F (2014) Improved vectors and genome-wide libraries for CRISPR screening. Nat Methods 11:783–784. 10.1038/nmeth.3047 25075903 PMC4486245

[B112] Sanson KR, Hanna RE, Hegde M, Donovan KF, Strand C, Sullender ME, Vaimberg EW, Goodale A, Root DE, Piccioni F, Doench JG (2018) Optimized libraries for CRISPR-Cas9 genetic screens with multiple modalities. Nat Commun 9:5416. 10.1038/s41467-018-07901-8 30575746 PMC6303322

[B113] Savell KE, Bach SV, Zipperly ME, Revanna JS, Goska NA, Tuscher JJ, Duke CG, Sultan FA, Burke JN, Williams D, Ianov L, Day JJ (2019) A neuron-optimized CRISPR/dCas9 activation system for robust and specific gene regulation. eNeuro 6 10.1523/ENEURO.0495-18.2019 PMC6412672 30863790

[B114] Sharon E, Chen SA, Khosla NM, Smith JD, Pritchard JK, Fraser HB (2018) Functional genetic variants revealed by massively parallel precise genome editing. Cell 175:544–557.e6. 10.1016/j.cell.2018.08.057 30245013 PMC6563827

[B115] Shmakov S, Smargon A, Scott D, Cox D, Pyzocha N, Yan W, Abudayyeh OO, Gootenberg JS, Makarova KS, Wolf YI, Severinov K, Zhang F, Koonin EV (2017) Diversity and evolution of class 2 CRISPR-Cas systems. Nat Rev Microbiol 15:169–182. 10.1038/nrmicro.2016.184 28111461 PMC5851899

[B116] Slaymaker IM, Gao L, Zetsche B, Scott DA, Yan WX, Zhang F (2016) Rationally engineered Cas9 nucleases with improved specificity. Science 351:84–88. 10.1126/science.aad5227 26628643 PMC4714946

[B117] Smith ZD, Meissner A (2013) DNA methylation: roles in mammalian development. Nat Rev Genet 14:204–220. 10.1038/nrg3354 23400093

[B118] Staahl BT, Benekareddy M, Coulon-Bainier C, Banfal AA, Floor SN, Sabo JK, Urnes C, Munares GA, Ghosh A, Doudna JA (2017) Efficient genome editing in the mouse brain by local delivery of engineered Cas9 ribonucleoprotein complexes. Nat Biotechnol 35:431–434. 10.1038/nbt.3806 28191903 PMC6649674

[B119] Sun JY, Anand-Jawa V, Chatterjee S, Wong KK (2003) Immune responses to adeno-associated virus and its recombinant vectors. Gene Ther 10:964–976. 10.1038/sj.gt.3302039 12756417

[B120] Swiech L, Heidenreich M, Banerjee A, Habib N, Li Y, Trombetta J, Sur M, Zhang F (2015) In vivo interrogation of gene function in the mammalian brain using CRISPR-Cas9. Nat Biotechnol 33:102–106. 10.1038/nbt.3055 25326897 PMC4492112

[B121] Tanenbaum ME, Gilbert LA, Qi LS, Weissman JS, Vale RD (2014) A protein-tagging system for signal amplification in gene expression and fluorescence imaging. Cell 159:635–646. 10.1016/j.cell.2014.09.039 25307933 PMC4252608

[B122] Thyme SB, Pieper LM, Li EH, Pandey S, Wang Y, Morris NS, Sha C, Choi JW, Herrera KJ, Soucy ER, Zimmerman S, Randlett O, Greenwood J, McCarroll SA, Schier AF (2019) Phenotypic landscape of schizophrenia-associated genes defines candidates and their shared functions. Cell 177:478–491.e20. 10.1016/j.cell.2019.01.048 30929901 PMC6494450

[B123] Tian R, Gachechiladze MA, Ludwig CH, Laurie MT, Hong JY, Nathaniel D, Prabhu AV, Fernandopulle MS, Patel R, Abshari M, Ward ME, Kampmann M (2019) CRISPR interference-based platform for multimodal genetic screens in human iPSC-derived neurons. Neuron 104:239–255.e2. 10.1016/j.neuron.2019.07.014 31422865 PMC6813890

[B124] Tzelepis K, Koike-Yusa H, De Braekeleer E, Li Y, Metzakopian E, Dovey OM, Mupo A, Grinkevich V, Li M, Mazan M, Gozdecka M, Ohnishi S, Cooper J, Patel M, McKerrell T, Chen B, Domingues AF, Gallipoli P, Teichmann S, Ponstingl H, et al. (2016) A CRISPR dropout screen identifies genetic vulnerabilities and therapeutic targets in acute myeloid leukemia. Cell Rep 17:1193–1205. 10.1016/j.celrep.2016.09.079 27760321 PMC5081405

[B125] Uezu A, Kanak DJ, Bradshaw TW, Soderblom EJ, Catavero CM, Burette AC, Weinberg RJ, Soderling SH (2016) Identification of an elaborate complex mediating postsynaptic inhibition. Science 353:1123–1129. 10.1126/science.aag0821 27609886 PMC5432043

[B126] Wang D, Tai PWL, Gao G (2019) Adeno-associated virus vector as a platform for gene therapy delivery. Nat Rev Drug Discov 18:358–378. 10.1038/s41573-019-0012-9 30710128 PMC6927556

[B127] Wang G, Chow RD, Ye L, Guzman CD, Dai X, Dong MB, Zhang F, Sharp PA, Platt RJ, Chen S (2018) Mapping a functional cancer genome atlas of tumor suppressors in mouse liver using AAV-CRISPR-mediated direct in vivo screening. Sci Adv 4:eaao5508. 10.1126/sciadv.aao5508 29503867 PMC5829971

[B128] Wang H, Yang H, Shivalila CS, Dawlaty MM, Cheng AW, Zhang F, Jaenisch R (2013) One-step generation of mice carrying mutations in multiple genes by CRISPR/Cas-mediated genome engineering. Cell 153:910–918. 10.1016/j.cell.2013.04.025 23643243 PMC3969854

[B129] Wang Y, Liu J, Huang BO, Xu YM, Li J, Huang LF, Lin J, Zhang J, Min QH, Yang WM, Wang XZ (2015) Mechanism of alternative splicing and its regulation. Biomed Rep 3:152–158. 10.3892/br.2014.407 25798239 PMC4360811

[B130] Williams A, Henao-Mejia J, Flavell RA (2016) Editing the mouse genome using the CRISPR-Cas9 system. Cold Spring Harb Protoc 2016:pdb.top087536 10.1101/pdb.top087536 PMC4898480 26832693

[B131] Wojno AP, Pierce EA, Bennett J (2013) Seeing the light. Sci Transl Med 5:175fs8. 10.1126/scitranslmed.3005798 23467559

[B132] Wu X, Scott DA, Kriz AJ, Chiu AC, Hsu PD, Dadon DB, Cheng AW, Trevino AE, Konermann S, Chen S, Jaenisch R, Zhang F, Sharp PA (2014) Genome-wide binding of the CRISPR endonuclease Cas9 in mammalian cells. Nat Biotechnol 32:670–676. 10.1038/nbt.2889 24752079 PMC4145672

[B133] Yang H, Wang H, Jaenisch R (2014) Generating genetically modified mice using CRISPR/Cas-mediated genome engineering. Nat Protoc 9:1956–1968. 10.1038/nprot.2014.134 25058643

[B134] Yarrington RM, Verma S, Schwartz S, Trautman JK, Carroll D (2018) Nucleosomes inhibit target cleavage by CRISPR-Cas9 in vivo. Proc Natl Acad Sci USA 115:9351–9358. 10.1073/pnas.1810062115 30201707 PMC6156633

[B135] Yeo NC, Chavez A, Lance-Byrne A, Chan Y, Menn D, Milanova D, Kuo CC, Guo X, Sharma S, Tung A, Cecchi RJ, Tuttle M, Pradhan S, Lim ET, Davidsohn N, Ebrahimkhani MR, Collins JJ, Lewis NE, Kiani S, Church GM (2018) An enhanced CRISPR repressor for targeted mammalian gene regulation. Nat Methods 15:611–616. 10.1038/s41592-018-0048-5 30013045 PMC6129399

[B136] Yin H, Xue W, Chen S, Bogorad RL, Benedetti E, Grompe M, Koteliansky V, Sharp PA, Jacks T, Anderson DG (2014) Genome editing with Cas9 in adult mice corrects a disease mutation and phenotype. Nat Biotechnol 32:551–553. 10.1038/nbt.2884 24681508 PMC4157757

[B137] Yin H, Kauffman KJ, Anderson DG (2017) Delivery technologies for genome editing. Nat Rev Drug Discov 16:387–399. 10.1038/nrd.2016.280 28337020

[B138] Zetsche B, Volz SE, Zhang F (2015) A split-Cas9 architecture for inducible genome editing and transcription modulation. Nat Biotechnol 33:139–142. 10.1038/nbt.3149 25643054 PMC4503468

[B139] Zheng Y, Shen W, Zhang J, Yang B, Liu YN, Qi H, Yu X, Lu SY, Chen Y, Xu YZ, Li Y, Gage FH, Mi S, Yao J (2018) CRISPR interference-based specific and efficient gene inactivation in the brain. Nat Neurosci 21:447–454. 10.1038/s41593-018-0077-5 29403034

[B140] Zhou H, Liu J, Zhou C, Gao N, Rao Z, Li H, Hu X, Li C, Yao X, Shen X, Sun Y, Wei Y, Liu F, Ying W, Zhang J, Tang C, Zhang X, Xu H, Shi L, Cheng L, et al. (2018a) In vivo simultaneous transcriptional activation of multiple genes in the brain using CRISPR-dCas9-activator transgenic mice. Nat Neurosci 21:440–446. 10.1038/s41593-017-0060-6 29335603

[B141] Zhou XX, Zou X, Chung HK, Gao Y, Liu Y, Qi LS, Lin MZ (2018b) A single-chain photoswitchable CRISPR-Cas9 architecture for light-inducible gene editing and transcription. ACS Chem Biol 13:443–448. 10.1021/acschembio.7b00603 28938067 PMC5820652

